# Comparison of Coastal Wetland Vegetation Assessment Methods in Southern California

**DOI:** 10.1007/s12237-026-01779-2

**Published:** 2026-07-20

**Authors:** Janet B. Walker, Rachel S. Smith, Kathryn M. Beheshti, Karina K. Johnston, Christine R. Whitcraft, Jeffrey A. Crooks, Melodie Grubbs, Henry M. Page, Steve Schroeter, Eric D. Stein, Kellie A. Uyeda

**Affiliations:** 1 Southern California Coastal Water Research Project, Costa Mesa, CA USA; 2 Marine Science Institute, University of California Santa Barbara, Santa Barbara, CA USA; 3 California State University, Long Beach, Long Beach, CA USA; 4 Tijuana River National Estuarine Research Reserve, Imperial Beach, CA USA; 5 Morro Bay National Estuary Program, Morro Bay, CA USA; 6 University of California San Diego, La Jolla, San Diego, CA USA

**Keywords:** Vegetation, Monitoring, Method comparison, Estuaries, Percent cover estimates

## Abstract

Vegetation surveys provide key information for evaluating functions within and across estuaries. However, survey methods and analyses are often highly variable, site-specific, and driven by monitoring program goals and objectives, making regional comparisons that include multiple programs difficult. This study compared multiple transect- and quadrat-based vegetation monitoring methods within eight southern California estuaries to assess variability and comparability among methods within programs and to provide recommendations for the development of regional and statewide coastal wetland monitoring programs. Using data from established monitoring programs with distinct, non-overlapping goals, vegetation percent cover, species richness, and community composition across five commonly used methods (line intercept, point contact, swath intercept, standard (visually assessed) quadrat, and laser quadrat) were compared. Of the seven vegetation cover comparisons made within programs (*n* = 2) and methods (*n* = 5), estimates did not vary by more than 7% on average, with the exception (12%) of the one newly restored and sparsely vegetated wetland included in the analysis. Similarly, plant community composition was not significantly different between methods. Whether the methods were comparable depended on three main factors: (1) method category (transect vs. quadrat), (2) the total amount of cover (high vs. low), and (3) the type of vegetation metrics (percent cover, species richness, and community composition). We also examined how survey decisions (e.g., bounding, plant state, nomenclature, binning) affected vegetation cover estimates within each monitoring program. Surprisingly, standardization across survey decisions was not key to the ability to compare within site estimates. However, it was critical for surveyors to first collect bounded vegetated and non-vegetated cover estimates (i.e., whether overall cover estimates can exceed 100%). When survey designs were similar (e.g., all transect-based and bounded), the data required less manipulation when combining and therefore had higher quality assurance than when survey designs varied (e.g. transect versus quadrat or bounded versus unbounded or combination). Review of method comparisons and survey decisions is reflected in a conceptual framework and led to six conclusions and recommendations for standardized, site-intensive vegetation monitoring that can inform broader recommendations for regional coastal wetland monitoring programs.

**Supplementary Information:**

The online version contains supplementary material available at 10.1007/s12237-026-01779-2.

## Introduction

Vegetation cover and species richness are among the most commonly surveyed indicators for coastal wetland monitoring programs, as vegetation is functionally associated with multiple ecosystem services (e.g., habitat for wildlife, primary production, carbon storage, water conservation, nutrient cycling, climate regulation; Dahlgren et al., 1997; Millennium Ecosystem Assessment, 2005; Godínez-Alvarez et al., 2009; Barbier et al., 2011; Lavorel, 2013). Vegetation is routinely surveyed in coastal wetlands to evaluate habitat degradation, climate impacts, propagation of invasive species, and restoration or recovery trajectories (Raposa et al., 2020; Watson et al., 2017). Coastal wetlands are increasingly subjected to the pressures of urban expansion, invasive species proliferation, management alterations, and the uncertainty associated with climate change (Cahoon et al., 2019; Pendleton et al., 2012; Thomsen et al., 2022; Watson et al., 2017). Robust records of vegetation communities are crucial to understanding ecosystem dynamics and projecting future wetland change (Seabloom & Valk, 2003; Vittoz & Guisan, 2007). However, inconsistent monitoring and assessment for vegetation cover in coastal wetlands pose a challenge for synthesizing data across monitoring programs that have distinct programmatic goals; these differences ultimately limit the ability to investigate regional and statewide patterns and trends. To understand and evaluate trends in vegetation communities at the statewide, regional, and nationwide levels, resource managers must understand how they can best consolidate vegetation datasets to effectively manage, preserve, and restore wetlands. Although recent advances in remote sensing allow for broad characterization of marsh extent, vegetation mapping, or vegetation indices in coastal wetlands (Dale et al., 2020), these methods (Alam & Hossain, 2021; Lopes et al., 2020) require validation through ground-based survey methods (Yeo et al., 2020), especially at new restoration sites with lower vegetation cover (Thomsen et al., 2022), and often lack the species-level data resolution of field surveys.

Ground-based vegetation surveys provide key information for evaluating coastal wetland functions within and across estuaries, but the implementation and analysis methods are highly variable and often site-specific (Raposa et al., 2020). Although collecting vegetation data is relatively rapid and inexpensive, inconsistent monitoring occurs with varied data collection protocols that are determined by program objectives, vegetation community type, and habitat heterogeneity (Floyd & Anderson, 1987; Godínez-Alvarez et al., 2009; Kent & Coker, 1992). Survey data with varying data protocols may be sensitive to site characteristics, biased by vegetation structure, or limited in reproducibility and accuracy due to inherent subjectivity (Helm & Mead, 2004; Kennedy & Addison, 1987; Vittoz & Guisan, 2007). Therefore, assessing the degree of comparability among vegetation survey methods is important for drawing inferences about spatial and temporal variability. For example, the National Estuarine Research Reserve System (NERRS) only adopted a consistent protocol for monitoring vegetation across the nation’s estuaries in the last decade (around 2010), and in 2021, formed a team, The National Marsh Synthesis Team (NAMSTE), to provide recommendations on integration of the individual reserve datasets into a standardized national data frame. These types of decisions are critical for the NERRS Program to systematically combine their historic datasets for comparison to those collected with the updated protocol (NERR 2025; Peter et al., 2020). However, a structured approach to combining disparate datasets that incorporates individual survey method biases is not readily available in the scientific literature. Sources and magnitudes of error are often not identified or error analysis is limited to individual studies (Kennedy & Addison, 1987).

Numerous methods have been developed to estimate the localized proportion of areal coverage (or percent cover) by different plant species within natural ecosystems (Floyd & Anderson, 1987). Methods to estimate vegetation percent cover can be broadly categorized as (a) transect-based methods (e.g., line-intercept via transects); (b) plot or quadrat-based methods (e.g., plots or quadrats); and (c) use of multi-spectral aerial imagery ground-truthed by point-contact estimates in band transects (Page et al., 2025). Variations in survey decisions, particularly for quadrat-based assessment methods (e.g., plot size, bounding cover, plant state, variations in total cover estimates, nomenclature, binning), complicates the identification of survey method limitations (Raposa et al., 2020). Inaccuracies within datasets collected at small scales can be magnified as transect-level data are extrapolated to characterize larger habitat areas. As accuracy, precision, and error can vary between methodologies, it is essential to evaluate different methods.

It is critical to understand how or if vegetation data can be combined or compared given disparate program objectives especially as many state and national monitoring programs become more established (e.g., The National Aquatic Resource Surveys (NARS) and Louisiana’s Coastwide Reference Monitoring System (CRMS), (Coastal Protection and Restoration Authority (CPRA) of Louisana, 2022; U.S. Environmental Protection Agency, 2024). For example, The National Wetland Condition Assessment (NWCA; part of the NARS) was designed to answer basic questions about the extent to which U.S. wetlands support healthy ecological conditions and the prevalence of key stressors at the national and regional scale (U.S. Environmental Protection Agency, 2024). In contrast to larger programs, regional monitoring and project-based monitoring may have different programmatic objectives (e.g., performance-based monitoring, (Page et al., 2022). Despite varying objectives, an evaluation of monitoring methods is necessary to progress towards a comprehensive statewide and nationwide monitoring strategy and to answer broader management questions. This project represents one effort to compare vegetation metrics of multiple vegetation monitoring methods in coastal wetlands within programs to assess variability and comparability among methods and provide recommendations for combining datasets.

The purpose of this study was to compare the results of multiple vegetation monitoring methods and decisions within three wetland monitoring programs that had non-overlapping monitoring goals, distinct management objectives, and variable levels of replication. The comparison and distinct goals were used to inform the development of a regional monitoring strategy and provide recommendations for consistency in the application of methods and comparison across datasets. Specifically, we identified key indicators (vegetation metrics, survey decisions) that differed among monitoring programs and then evaluated the following research questions to provide recommendations that can be extended to regional scale monitoring:

1. How do vegetation metrics (percent cover, species richness, community composition) compare across methods within three wetland monitoring programs?
2. How do survey decisions (bounding, nomenclature, plant state, binning) affect vegetation percent cover estimates within three wetland monitoring programs?

## Methods

### Study Region

**Fig. 1:**
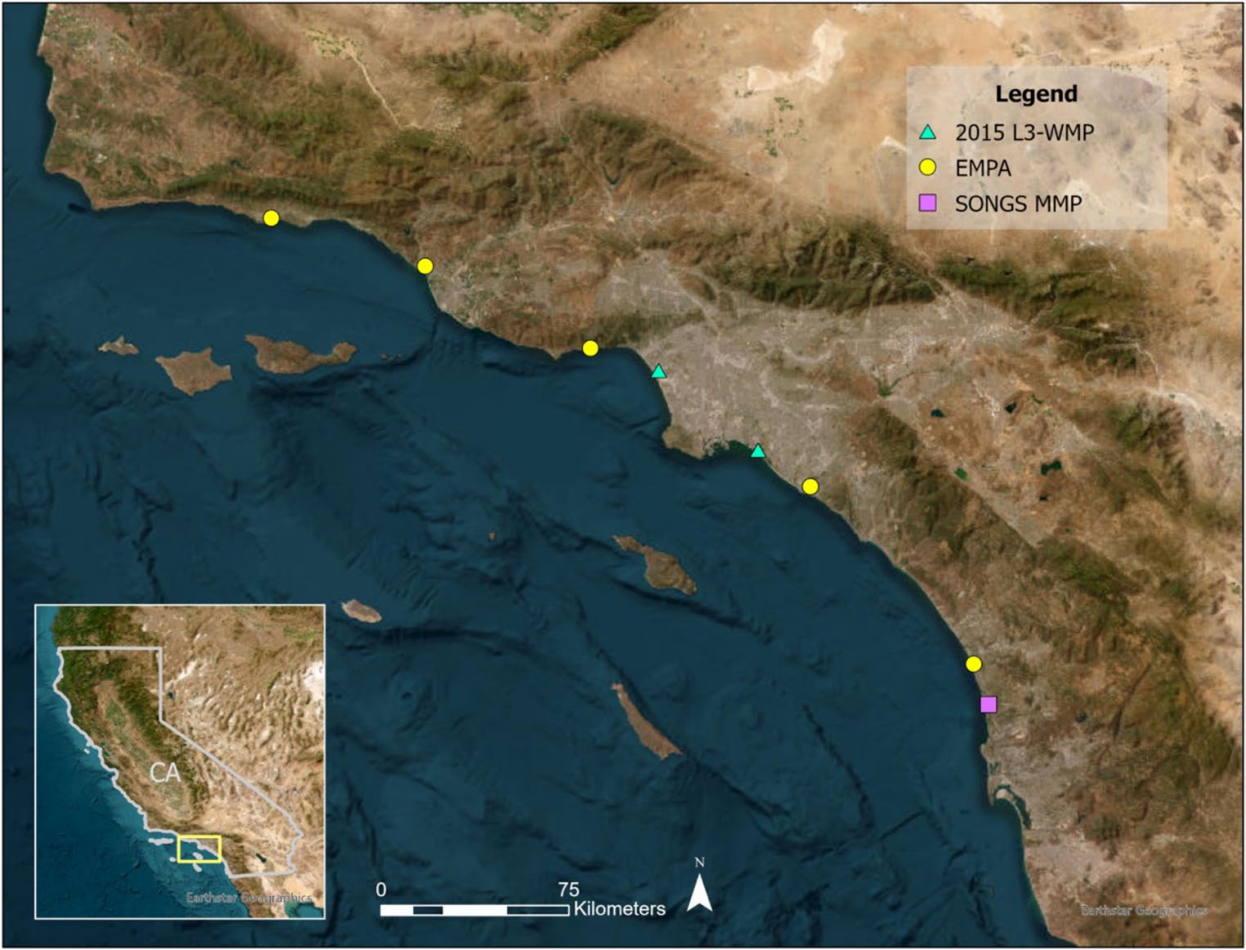
Map of southern California coastal monitoring sites. Symbols represent locations of three monitoring programs: the 2015 Level 3 Wetland Monitoring Pilot (L3-WMP; triangle), the Estuarine Marine Protected Area (EMPA; circle), and the San Onofre Nuclear Generating Station Mitigation Monitoring Program (SONGS MMP; square). Insets show the location of the study region within California. Scale bar and north arrow are provided for reference

Southern California’s coastal wetlands range from large open embayment estuaries to intermittently closed estuaries (SCWRP, 2018). Southern California estuaries are present in an arid to semi-arid, Mediterranean climate with mild, wet winters and hot, dry summers. This seasonal pattern affects estuarine hydrology (e.g., tidal inundation, freshwater inputs, and estuarine circulation) and salinity, leading to unique ecological dynamics, flora, and fauna. Many southern California estuaries are close to major urban centers, and stressors include development, hydrological alterations, pollution, and others. These stressors impact wetland ecological health and necessitate targeted conservation, management, and monitoring efforts. Although each system is unique, there are underlying environmental similarities in watershed size, morphology, and mouth dynamics among estuaries that influence resident biota within typologies, or wetland archetypes. In 2020, regional expert panels were assembled to assign regional estuarine condition scores, but early in the process, experts realized that program-specific datasets used non-comparable or disparate methods that made regional-scale analyses complex and infeasible without first evaluating within-program variations. To address the research questions described above, we compiled data from three programs using five different vegetation survey methods across eight southern California coastal wetlands (Fig. 1).

### Monitoring Programs

We used data collected from three different programs: the 2012–2015 Level 3 Wetland Monitoring Program (2015 L3-WMP), San Onofre Nuclear Generating Station Mitigation Monitoring Program (SONGS MMP), and California Estuary Marine Protected Area Monitoring Program (EMPA). Each program has extensive history in monitoring and assessing southern California wetlands; however, each has distinct motivations for collecting vegetation data. The 2015 L3-WMP, led by The Bay Foundation (TBF), was a regional Level 3 (site-intensive) wetland monitoring program funded by United States Environmental Protection Agency to assess the condition of southern California wetlands (Johnston et al., 2015) and develop Level 3 monitoring protocols (Johnston et al., 2020, 2021). The SONGS MMP program, led by the University of California Santa Barbara, monitors and evaluates the performance of a restored wetland. The restored 60 ha wetland serves as out-of-kind mitigation to compensate for fish losses throughout the Southern California Bight caused by SONGS operations. Field-based vegetation surveys are not part of the required compliance monitoring of the SONGS MMP, but vegetation sampling was conducted to evaluate vegetation cover wetland-wide within the mid to high marsh zone to corroborate aerial imagery data collected as part of compliance monitoring and to track the performance of a large-scale planting effort (Page et al., 2025). The EMPA monitoring program, funded by the Ocean Protection Council and co-led in southern California by the Southern California Coastal Water Research Project and California State University, Long Beach, is an ongoing effort to assess the quality and condition of estuaries statewide with a focus on Marine Protected Areas (MPAs). The program goals are to monitor California estuaries with a standard, comprehensive function-based assessment framework to determine the health of California’s estuaries and the efficacy of MPA designation.

The programs measured a diverse set of wetland types across about 300 km of coastline. The 2015 L3-WMP and SONGS MMP focused on perennially open systems, while the EMPA program monitored a diversity of wetland archetypes, including large embayments, intermittently open systems, and river valley systems. The 2015 L3-WMP includes two sites. One site is 233 ha perennially open estuary that is highly degraded and disturbed with severe hydrological impacts and restrictions (e.g., concrete levees, culverts, bisecting roads) due to urbanization. Much of the site has been impacted by historic fill placement and type conversion of wetlands to impacted upland habitats primarily dominated by invasive plants. The second site is a large wetland complex spanning approximately 971 ha; however, most of the historic wetlands have been filled, lost, or degraded due to construction of nearby power plants. Both sites are tidally open with expansive salt marsh habitat. The SONGS MMP monitors a river-valley estuary, which underwent restoration to preserve, improve, and create a variety of habitats to increase and maintain fish and wildlife, ensure the protection of endangered species, and ensure adequate tidal and riverine flushing and circulation to support a diversity of biological resources while maintaining the appearance of a natural wetland ecosystem. The site is tidally open with expansive *Spartina foliosa* (Pacific cordgrass) habitat. The EMPA program monitors five sites, which span a diversity of wetland types. Three of the sites are temporarily closed systems and therefore experience event-driven mouth closures impacting the tidal regime and surrounding habitat. The other two sites are permanently open lagoon systems with open tidal prisms and expansive salt marsh and bird habitat.

### Survey Methods

**Fig. 2:**
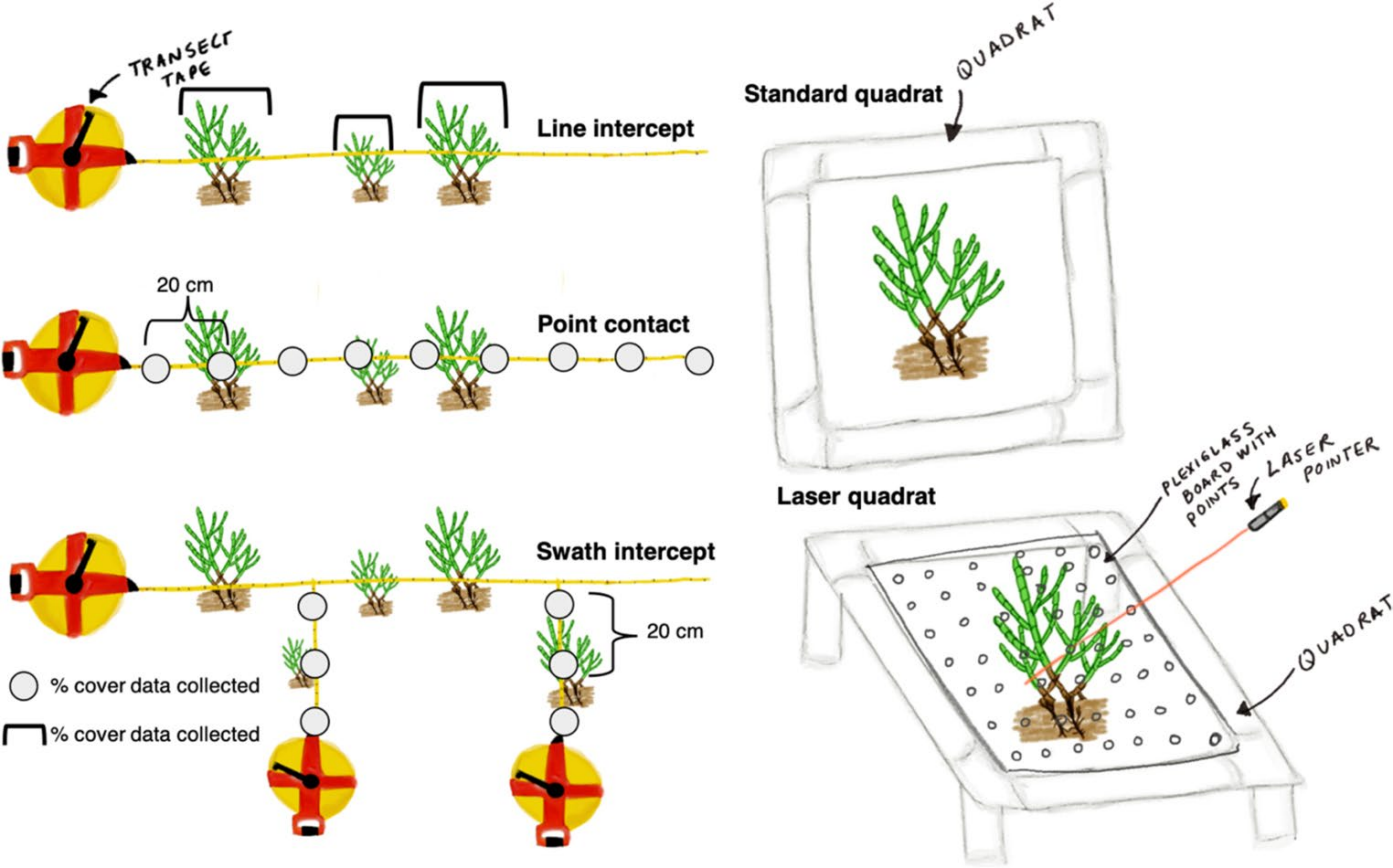
Drawings of survey methods. Methods were subdivided into two categories: transect and quadrat. Transect methods are measurements made along a single transect tape or line. These methods do not use quadrats or plots to make cover estimates. We include three transect methods in this comparison: line intercept, point contact, and swath intercept. Quadrat methods estimate cover and vegetation composition within a confined plot or quadrat. Typically, quadrats are placed along transect lines at equal or random intervals to establish the monitoring plots. We include two quadrat methods: standard and laser

For the programs that measured vegetation cover with multiple methods concurrently (2015 L3-WMP, SONGS MMP), we compared methods that survey vegetation composition and cover within each program (Table 1. Figure 2). We subdivided methods into two categories: transect and quadrat. Transect methods are measurements made along a single transect tape or line. These methods do not use quadrats or plots to make cover estimates. We include three transect methods in this comparison: line intercept, point contact, and swath intercept. Quadrat methods estimate cover and vegetation composition within a confined plot or quadrat. Typically, quadrats are placed along transect lines at equal or random intervals to establish the monitoring plots. We include two quadrat methods: standard and laser. For detailed drawings of each method, see Fig. 2.

**Table 1:** Comparative descriptions of transect- and quadrat-based vegetation cover methods. Description of the transect-based and quadrat-based vegetation cover methods, including (A) line intercept, (B) point contact, (C) swath intercept, (D) standard quadrat, and (E) laser quadrat, relevant survey decisions (bounding, nomenclature, plant state, and binning), as well as the programs that use each method (2012–2015 Level 3 Wetland Monitoring Program (2015 L3-WMP); San Onofre Nuclear Generating Station Mitigation Monitoring Program (SONGS MMP); and the California Estuary Marine Protected Area Monitoring Program (EMPA))

	Transect			Quadrat	
	A. Line intercept	B. Point contact	C. Swath intercept	D. Standard	E. Laser
Description	Record every species observed directly below the transect tape where the vegetation crosses a minimum length (e.g., > 1 cm).	Record vegetated and non-vegetated observation below the transect tape at a set distance between points (e.g., every 20 cm).	At a regular interval along a main transect tape, position a shorter transect perpendicular to the main transect line and record every species observed below the perpendicular transect tape at a set distance between points (e.g., every 50 cm)	Record percent cover (raw or Daubenmire cover class) within the quadrat area.	Insert a laser pointer into 49 evenly distributed points on a Plexiglas board and record the species that the laser hits first.
Relevant survey decisions	Nomenclature Plant state	Nomenclature Plant state	Nomenclature Plant state	Bounding Nomenclature Plant State Binning	Nomenclature Plant State
Data source	2015 L3-WMP (*n* = 2 sites)	2015 L3-WMP (*n* = 2 sites)	SONGS MMP (*n* = 1 site)	2015 L3-WMP (*n* = 2 sites), SONGS MMP (*n* = 1 sites), EMPA (*n* = 5 sites)	2015 L3-WMP (*n* = 2 sites)

#### Transect Methods

For the line-intercept method, surveyors record every species observed directly below the transect tape (Table 1A). Surveyors walk along a transect tape (transect length may vary) and every time the transect tape intersects or crosses with a plant species, the surveyor records the species observed. Typically, surveyors establish a minimum length between each vegetation-tape intersection (e.g., > 1 cm).

In contrast, for the point contact method, surveyors record every species or cover type (e.g., bare cover, wrack, algae, etc.) observed below the transect tape at a predetermined set distance between observations (e.g., every 20 cm; Table 1B). For the swath intercept method, surveyors establish a shorter, secondary transect tape, or “swath”, perpendicular to the primary tape at regular intervals (Table 1C). Surveyors then record every species observed below the perpendicular transect tape at a set distance between points (e.g., every 50 cm). For both methods, surveyors only record the plant species or cover type that physically touch the transect tape.

Detailed methods for the line-intercept and point contact methods are included in Page et al. (2025) and Johnston et al. (2021), Appendix B, Standard Operating Procedure 3.2: Vegetation Cover Surveys.

#### Quadrat Methods

Both the standard (visually assessed) quadrat and laser quadrat methods estimate the percent cover of the plant species within designated plots or quadrats of known area. The standard quadrat method may use overall visual estimates of cover by species (Table 1D). Quadrats are often divided into squares to help surveyors visualize the estimated cover of each species. For example, if quadrats are 1 m^2^, then the quadrat could be divided into 16 equal square pieces (or a 10 × 10 grid for higher resolution). In contrast, the laser method uses distributed points to identify the plant species throughout the plot (Table 1E). For example, the quadrats used for the laser method in our study included 49 evenly distributed points (or holes) on a plexiglass board. A laser pointer was directed through each hole and the species and cover types that the laser hit first were recorded.

Detailed methods for the standard (visually assessed) quadrat method are outlined in EMPA (2024), specifically the Standard Operating Procedure #11. Detailed methods for the laser method are included in Johnston et al. (2021), Appendix B, Standard Operating Procedure 3.2: Vegetation Cover Surveys.

### Survey Decisions

Monitoring programs make several decisions when classifying and estimating vegetation cover. These decisions include whether to (1) assess bounded vs. unbounded cover (e.g., whether the sum of individual vegetated and non-vegetated components together is “bounded” at 100% or can exceed 100%), (2) quantify percent cover of non-vegetated cover types into predetermined categories (“nomenclature”), (3) categorize vegetation by live and dead state (“plant state”), and (4) quantify percent cover in pre-determined bins versus estimating exact cover (“binning”). All methods require decisions related to nomenclature and plant state, and the standard quadrat method also requires decisions related to bounding and binning (Table 1). We explain those decisions below.

#### Bounding: Bounded vs. Unbounded Cover Estimates

When using the standard quadrat method, monitoring programs must determine whether overall cover estimates can exceed 100%, and long-standing regulatory monitoring programs have used both techniques (e.g., U.S. Army Corps of Engineers, 2010; U.S. Department of Agriculture & U.S. Deperatment of the Interior, 1996). Cover can either be assessed as (1) bounded cover, where individual vegetated and non-vegetated components together equal 100% cover or (2) unbounded cover, where individual vegetated and non-vegetated components together can exceed 100%. In the first case, percent cover is assessed using a birds-eye view (aerial estimate of the visible surface layer) that does not account for overlapping vegetated and non-vegetated layers. The second case allows for layering and overlap among species with different canopy heights. In both cases, surveyors can distinguish species-specific contributions to percent cover, although unbounded measures may more accurately reflect true cover by species and may be prioritized for species-level analyses, especially when numerous or complex canopy layers are present. Alternatively, bounded vegetation cover may correlate better with aerial imagery cover classifications and provide more consistent measures of unvegetated cover. Thus, the decision to bound data depends on the research question. In this study, we do not compare species-specific estimates and instead focus on total vegetated cover.

A critical metric to for comparison is total vegetated cover (i.e., the area occupied by vegetative cover of any type, analogous to what would be derived from most remote sensing approaches). However, when programs use different approaches to estimate cover (e.g., bounded vs. unbounded), adjustments are needed to derive a consistent metric. One strategy is to adjust the unbounded vegetated estimates by subtracting the amount of non-vegetated cover from 100% (total adjusted vegetation = 100 - non-vegetated cover). This approach allows comparisons of the total adjusted vegetation cover across methods. However, this adjustment requires that the sum of the individual components of non-vegetated cover (however assessed) does not exceed the overall area of non-vegetated cover (i.e., it is “bounded”).

Among our three programs, each program quantified vegetated and non-vegetated cover differently. The 2015 L3-WMP program estimated unbounded vegetated cover (vegetation > 100%) and bounded non-vegetated cover (non-vegetated cover ≤ 100%). The SONGS program bounded both vegetated and non-vegetated cover. Therefore, both programs could calculate an adjusted vegetation cover metric. The EMPA program did not bound vegetated or non-vegetated cover, making it impossible to calculate an adjusted vegetation metric.

#### Nomenclature: Non-Vegetated Cover Types

 For all methods, monitoring programs must also decide how to estimate the cover of non-vegetated areas within the quadrats. A common discrepancy among data is the nomenclature used to define non-vegetated space within the quadrat. Some surveyors define non-vegetated space as ‘bare’ or ‘open’ cover. Some surveyors create more categories to further define the type of cover within the non-vegetated space (e.g., rocks, algae, woody debris, mud, burrows). Standardizing nomenclature is important if surveyors ultimately want to compare specific categories across programs and methods.

 Multiple non-vegetated cover types can be added together to determine the estimated non-vegetated area, although it is important to also define whether overall estimates are bounded or unbounded. If non-vegetated estimates are bounded, then regardless of the number of categories, total non-vegetated estimates can be compared across programs (e.g., 2015 L3-WMP, SONGS). However, the EMPA program did not bound non-vegetated cover, which highlights the importance of decisions about non-vegetated cover type nomenclature. The EMPA program included a ‘bare’ category defined as any open space that was not vegetated cover (this category could not exceed 100%), as well as additional classes of cover (wrack, algae, trash, etc. that could exceed 100%). In these instances, it may be possible to calculate an adjusted vegetated cover by subtracting bare cover from 100 (total adjusted vegetation = 100 - bare). We tested the validity of this approach by comparing other cover classes within the non-vegetated category to the bare subcategory for the EMPA program.

#### Plant State: Live vs. Dead

For all methods, monitoring programs can choose to measure vegetation cover by either separating live and dead plant states or pooling live and dead plant states together. For example, if a plot has 50% cordgrass, composed of 40% live cordgrass and 10% dead cordgrass, one surveyor may state that there is 40% live cordgrass and 10% dead cordgrass on the datasheet, while another surveyor may state that there is 50% cordgrass. An important caveat is that surveyors may define ‘dead’ vegetation differently, as well as classify dead vegetation into either vegetated or non-vegetated classes. For all programs that distinguished between live and dead, dead was defined as intact, decaying plant stems. These plants are typically, but not always, brown. Senescing plants are typically classified as ‘live’. In our analyses, ‘dead’ vegetation is always classified as vegetated cover, not non-vegetated cover.

#### Binned Cover

When using the standard quadrat method, monitoring programs must decide whether to estimate percent cover of each species with pre-determined bins (rounded estimates) or by providing a specific cover value. Predetermined bins could be equally spaced bins or programs could choose to use established uneven binning methods, such as the Daubenmire (Daubenmire, 1959) or Braun-Blanquet (Braun-Blanquet, 1925) methods (described in detail below). For example, if a plot has 47% cordgrass, one surveyor may estimate a cover of 47%, while another surveyor may estimate it at a rounded 50% cover, depending on the pre-established bins.

### Program Survey Methods

Each program provided data collected using different survey methods and decisions to address program-specific objectives. Below we outline the specifics of the survey method used by each group, including survey location, year, and unit of replication.

#### 2015 L3-WMP Dataset

In 2013 and 2014, the 2015 L3-WMP conducted a field survey at two sites, specifically to compare cover estimates using the line intercept, point contact, standard quadrat, and laser quadrat methods measured concurrently at the same locations. Within each site, specific habitats were targeted to conduct all the methods, with a focus on vegetated marsh habitats. Transects were 30 m in length. All four survey methods were applied (i.e., line intercept, point contact, standard quadrat, and laser quadrat) on each established transect (*n* = 30). For both standard and laser quadrat methods, seven quadrats were surveyed on each transect.

All plant species were categorized as either live or dead plants when measuring percent cover. Cover for the standard quadrat method was estimated using the Daubenmire method, estimating cover on select bins: 0–1, > 1–5, > 5–25, > 25–50, > 50–75, > 75–95, and > 95–100% (detailed methods available in Johnston et al., 2020, 2021). Analyses were subsequently conducted by assigning the upper limit of each bin to all values located within that bin (e.g., 23% was assigned to the > 5–25 bin and assigned a value of 25%). For all transects, if the contact or quadrat cover was not plant tissue, the ground or cover type was recorded as non-vegetated which included bare, trash, wrack, or woody debris. Trash was defined as man-made debris, and wrack was defined as dead organic material such as kelp. Algae on top of plants was noted as present for each location, removed to reveal the plant tissue below, and not included in percent cover estimates; *Cuscuta pacifica* (salt marsh dodder) was recorded similarly. Because canopies with different growth strata may have overlapping layers and the cover is broken down into classes, the standard quadrat cover estimates may total more than 100% (Ambrose & Diaz, 2008), unlike the laser-based quadrat cover estimates, line intercept, and point contact methods, which never exceeded 100%. For the standard quadrats, total vegetated cover was evaluated by subtracting non-vegetated estimates from 100 (total adjusted vegetation = 100 - non-vegetated cover).

#### SONGS MMP Dataset

The SONGS MMP collected vegetation data using the standard quadrat and swath intercept methods at a restored, sparsely vegetated wetland. Surveyors established 48 transects in intertidal salt marsh, spanning elevations from 1.71 to 2.01 m NAVD88. Transects varied in length from 5 to 130 m, depending on wetland slope and extent. Vegetation was surveyed annually in 2012–2013. The standard quadrat and swath intercept methods were performed at the upper (2.01 m NAVD88) and lower elevation bound (1.71 m NAVD88) of each transect. For the swath intercept method, a 2 m pole marked in 50 cm intervals was centered perpendicularly on the main transect. Surveyors identified what was present under each 50 cm interval (species-level vegetation, non-vegetated) along each “swath” (*n* = 5 measurements per swath; *n* = 2 swaths per transect). Non-vegetated categories included bare, algae, wrack, debris, detritus, and wood.

For the standard quadrat method, visual estimates of percent cover were taken in two replicate 0.25 m^2^ quadrats at each elevation (*n* = 4 quadrats per transect). In 2013, in addition to the transect endpoints, surveyors collected data with both methods at additional 5-meter increments along each transect. In the standard quadrats, the SONGS MMP program estimated vegetated cover (upright, rooted plants) and non-vegetated cover. For vegetated cover, surveyors estimated the exact cover by species, distinguishing between live and dead state. Overall cover estimates (vegetated, non-vegetated) did not exceed 100%.

#### EMPA Dataset

In 2021, the EMPA used the standard quadrat method to conduct vegetation surveys at five sites in southern California. Surveyors established either six or nine 25 m transects in each coastal wetland, depending on the size of the wetland. Surveyors estimated the vegetation cover within 1 m^2^ quadrats every 5 m along the transect (*n* = 5 quadrats per transect). Three transects were surveyed within three vegetation zones: low, mid, and high. The vegetation zones were roughly established by setting transects in distinct elevation zones. Vegetation was surveyed in the spring and fall of 2021.

The EMPA program estimated the cover of each plant species and cover type to the nearest 1% (the ‘exact’ cover). In these surveys, both the vegetated cover and non-vegetated cover were allowed to exceed 100%. Surveyors estimated the percent cover by species, distinguishing between live and dead species as separate estimates of cover. Because canopies of different strata (e.g., grasses, shrubs) may overlap, cover estimates of individual species could total more than 100%. In 2021, the EMPA program did not limit the amount of non-vegetated cover type categories; therefore, surveyors could create their own categories of cover (bare, rock, wood, etc.). The EMPA program has since changed their methods due to the many lessons learned.

### Data Analysis

#### Methods Comparison

Two programs (2015 L3-WMP, SONGS MMP) used multiple methods to assess vegetation cover and species richness at the same time and location. To assess comparability among methods within each program, we fit a linear model for each possible combination of methods collected concurrently for each program. Linear regressions were chosen to visually evaluate estimations of cover or species richness between two methods against a 1:1 line. For the 2015 L3-WMP, we compared how point contact, line intercept, standard quadrat, and laser quadrat methods estimated vegetation cover and species richness. We fit six linear models for each combination of methods (point contact vs. line intercept, point contact vs. standard quadrat, etc.) for each vegetation metric (vegetation cover, species richness). For the standard quadrat data, if data were allowed to exceed 100% (2015 L3-WMP), we adjusted the unbounded data as described above (100 - non-vegetated) so that all methods were comparable on the same scale. For the SONGS MMP, we compared swath intercept and standard quadrat methods, fitting one linear model for each response variable. For all models, we examined model residuals and model diagnostics to assess model assumptions (Hartig, 2002).

For both programs, we used the inflection point to evaluate the percent cover value at which one method’s estimation of cover was similar (within 10%), under- or over-estimating cover relative to another method. We defined the inflection point as the point where the linear regression line from our data intersected the 1:1 line, which would indicate an exact data match. We identified the inflection point using the ggplotly function in the plotly package in R (Sievert, 2020). The inflection point was noted and the average difference in cover above and below the inflection point calculated for each combination of methods compared. At the inflection point, percent cover estimates should be most comparable while below or above the inflection point, estimates may converge or diverge as cover decreases or increases, respectively. Such an assessment has practical applications to wetland managers that may assess marsh condition by comparing vegetation cover over time using a dataset with inconsistent methods. For example, if an inflection point occurs when percent cover is 60%, and beyond 60%, estimates between methods converge, managers could assume that the data are comparable through time despite variable methods if overall cover typically exceeds 60%.

To assess differences in vegetation community composition by method for the two programs (2015 L3-WMP and SONGS MMP), we calculated separate pair-wise Bray-Curtis dissimilarity matrices and used permutational multivariate ANOVA (PERMANOVA) to examine how community composition varied by method (Oksanen et al., 2022). We used non-metric multidimensional scaling (NMDS) to visualize a two-dimensional ordination of community vegetation cover assessed with each method for both programs.

#### Survey Decisions

##### Bounding

Although we expected unbounded estimates to exceed vegetation cover compared to adjusted vegetation cover, we compared the adjusted unbounded vegetation cover estimates for 2015 L3-WMP (adjusted vegetation cover = 100 - non-vegetated) and EMPA (adjusted vegetation cover = 100 - bare) programs to the unbounded data within each program to determine if there were any noticeable patterns. We fit linear models for each program, with unbounded vegetation cover examined as a function of adjusted unbounded vegetation cover.

##### Nomenclature

To determine if calculations of adjusted vegetation cover are comparable when calculated using the bare versus bare + non-vegetated categories, we evaluated whether the other cover classes within the non-vegetated category are significantly different from the bare category (i.e., is bare cover similar to total non-vegetated cover?). We fit separate linear models for each site for the EMPA programs (*n* = 5). We used percent cover data collected from standard quadrats as the response and non-vegetated sub-groupings (bare, non-vegetated) as the main effect.

##### Plant state

Monitoring programs must also decide whether to discern between live and dead plant states vs. estimating live and dead plant states together for a particular species. To assess whether pooling live and dead plant states together is comparable to using live plant states alone, we fit separate linear models for each site for the SONGS MMP (*n* = 1) and EMPA programs (*n* = 5). We used percent cover data collected from standard quadrats as the response and plant status (live, live + dead) as the main effect.

##### Binning

Lastly, we assessed outcomes for vegetation cover data collected in standard quadrats using predetermined bins relative to unbinned cover. To assess comparability of different binning approaches relative to unbinned vegetation cover, we fit separate linear models for each site for the EMPA (*n* = 5) and the SONGS MMP (*n* = 1) programs. We binned data using equally spaced bins with different numbers of bins (5, 6, 9, and 11 groups) and using the Daubenmire (0–1, > 1–5, > 5–25, > 25–50, > 50–75, > 75–100, Daubenmire, 1959) and Braun-Blanquet methods (0–5, > 5–25, > 25–50, > 50–75, > 75–95, and > 95–100; Braun-Blanquet, 1925), two widely used, uneven binning methods. For all binning approaches, we assigned the upper limit of each bin to all values located within that bin. Each model included percent cover data as the response and binning method as the main effect. We used Tukey’s HSD post-hoc tests to evaluate differences (α < 0.05) between binning methods.

All data analyses were conducted using R, version 4.4.0 (R Core Team, 2024). For all models described, we examined model residuals and model diagnostics to assess model assumptions using the DHARMa package (Hartig, 2002).

## Results

### Methods Comparison

**Fig. 3:**
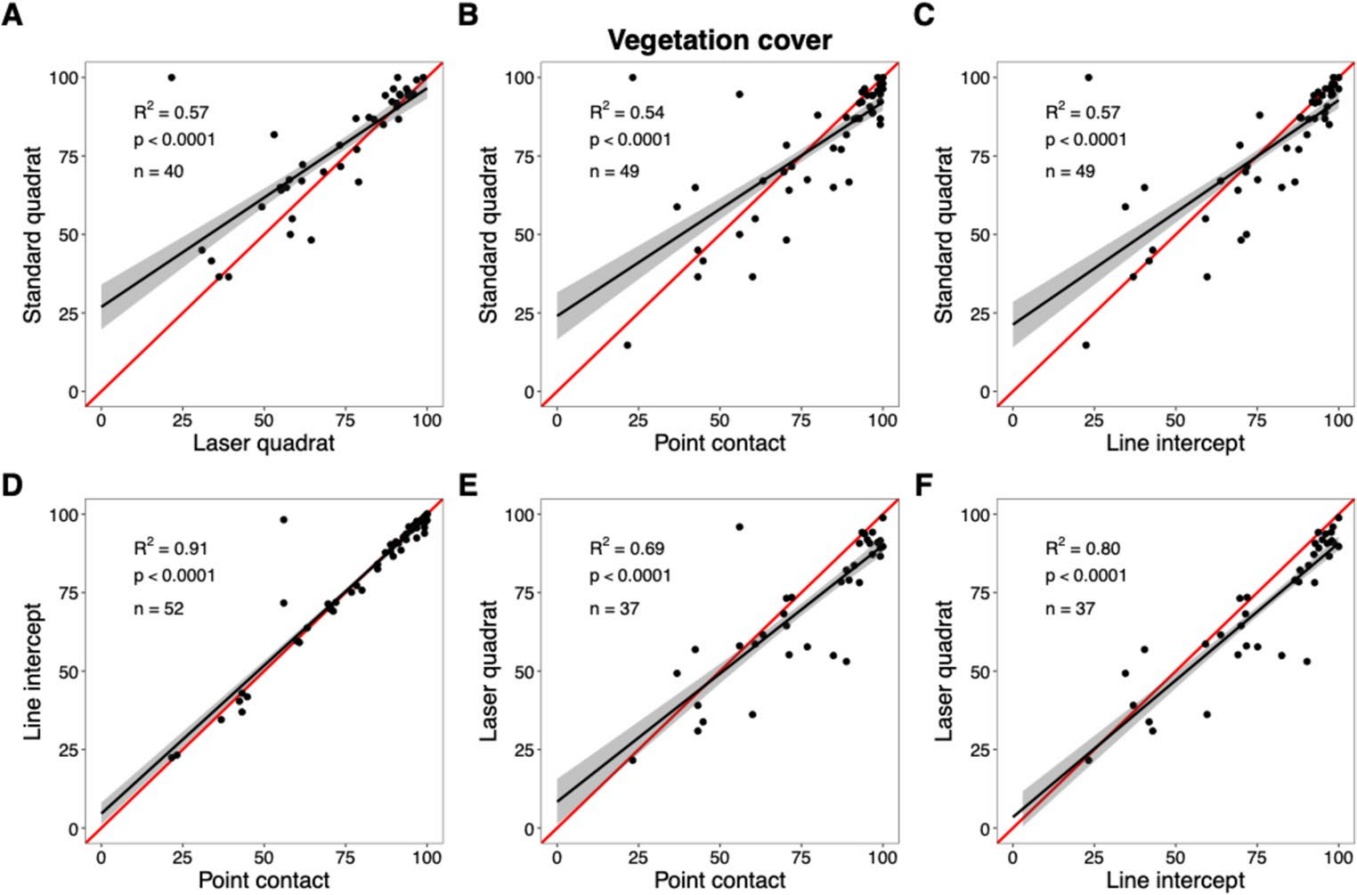
Methods comparison of total vegetation cover for 2015 L3-WMP. Total vegetation cover estimates comparing standard quadrat to (**A**) laser quadrat, (**B**) point contact, and (**C**) line intercept and comparing (**D**) line intercept to point contact, (**E**) laser quadrat to point contact and (**F**) laser quadrat to line intercept. Points represent vegetation cover estimates from the same plot using each method, the fitted regression line includes the 95th % confidence interval, and the fit of the line (R^2^), the associated p-value, and the sample size (n) are listed in the top left corner of each plot. The red line indicates the 1:1 line. For the standard quadrats, vegetation cover refers to the total adjusted vegetation cover (100% cover - non-vegetated cover)

Estimates of vegetation cover and species richness varied across methods and within monitoring programs. For the 2015 L3-WMP program, there were six method comparisons of vegetation cover (Fig. 3). Within those comparisons, the largest difference in estimates of vegetation cover relative to the 1:1 line was ~ 40%, with the exception of one outlier (~ 75%). The greatest agreement in vegetation cover estimates between methods occurred as cover increased and approached 100%, as the linear model fit approached the 1:1 line. Generally, standard quadrat methods estimated higher cover as compared to laser quadrat (Fig. 3A), when relative vegetation cover was less than ~ 75%. There was strong agreement between standard quadrat, point contact, and line intercept when vegetation cover was greater than 75%, as the standard error decreased, and the linear model fit approached the 1:1 line (Fig. 3A-C). However, outliers occurred as exceptions to the agreement trend, even at higher cover. There was strong agreement in estimates of vegetation cover between the similar survey protocols of point contact and line intercept methods (Fig. 3D; R² = 0.91). Point contact and laser quadrat (Fig. 3E) methods produced roughly similar estimates of vegetation cover, but there was considerable spread in the data with multiple instances where one method either over- or under-estimated cover relative to the other; comparisons between line intercept and laser quadrat (Fig. 3F) produced similar results.

The inflection point was used to evaluate the percent cover value at which one method’s estimation of cover was similar. Across all the methods compared, the average difference in total vegetation cover above and below the inflection point did not exceed 12% and was as low as 0.3% (Table 2). Most of the 2015 L3-WMP methods compared had inflection points where total vegetation cover was at or above 50%, with the exception of laser quadrat vs. line intercept (40%; Table 2). In most comparisons, the average difference in total vegetation cover was greater under the inflection point as opposed to above the inflection point where cover estimates between methods converged.

**Table 2:** Average difference in total vegetation cover estimates above and below inflection points for all methods compared. Reported differences in total vegetation cover between methods is the absolute average difference in mean cover under or above the inflection point. Inflection points were assessed by identifying where each linear fit (Figs. 3 and 5) intersected the 1:1 line

Program	Methods compared	Inflection point	Average difference in total vegetation cover under inflection point	Average difference in total vegetation cover above inflection point
SONGS	Standard quadrat vs. Swath intercept	20%	12%	2%
L3-WMP	Standard quadrat vs. Laser quadrat	80%	7%	3%
L3-WMP	Standard quadrat vs. Point contact	75%	0.6%	2%
L3-WMP	Standard quadrat vs. Line intercept	75%	1%	2%
L3-WMP	Line intercept vs. Point contact	60%	0.3%	1.3%
L3-WMP	Laser quadrat vs. Point contact	50%	6%	7%
L3-WMP	Laser quadrat vs. Line intercept	40%	3%	6%

**Fig. 4:**
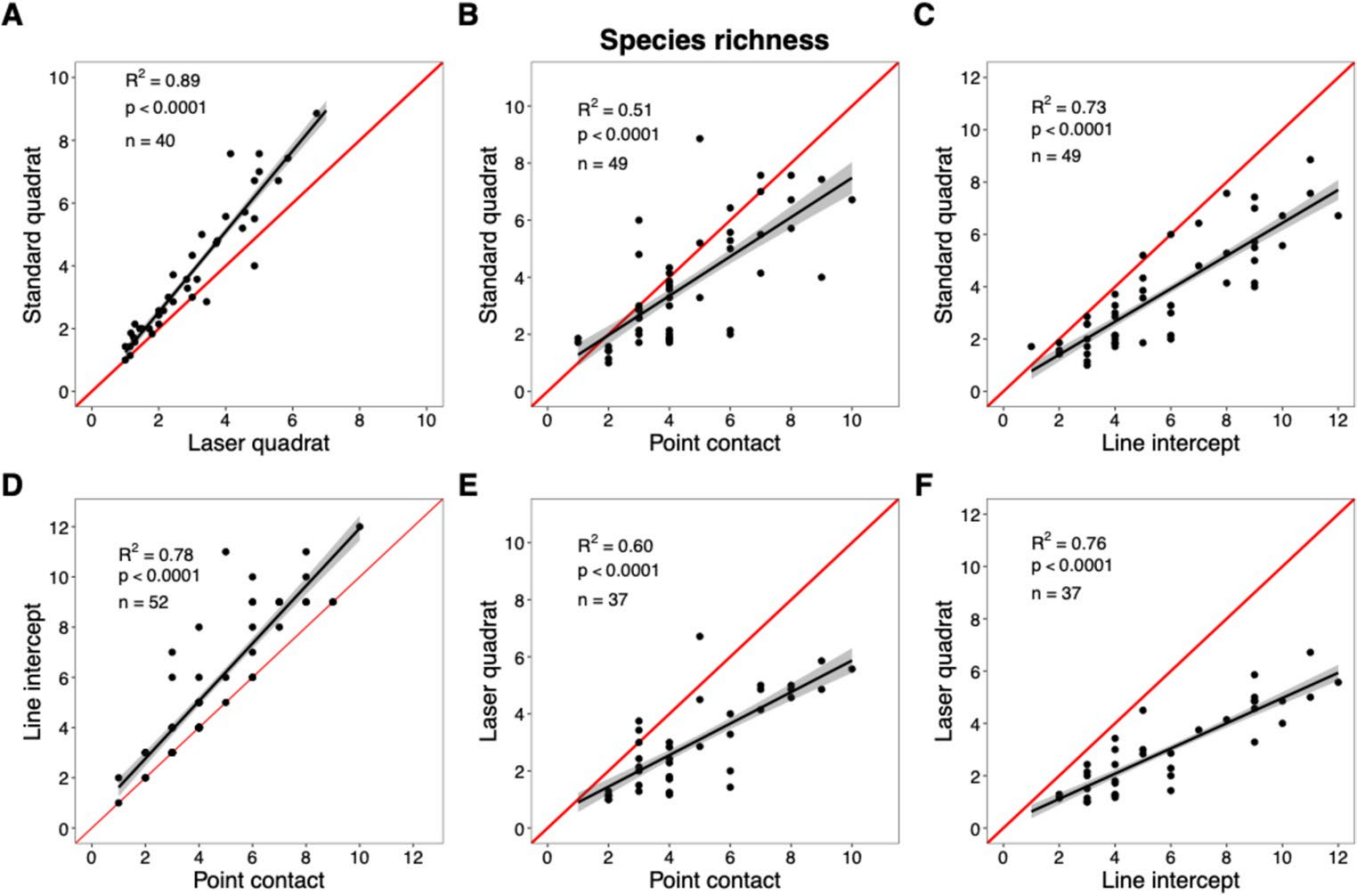
Methods comparison of species richness for 2015 L3-WMP. Species richness estimates comparing standard quadrat to (**A**) laser quadrat, (**B**) point contact, and (**C**) line intercept and comparing (**D**) line intercept to point contact, (**E**) laser quadrat to point contact and (**F**) laser quadrat to line intercept. Points represent vegetation cover estimates from the same plot using each method, the fitted regression line includes the 95th % confidence interval, and the fit of the line (R2), the associated p-value, and the sample size (n) are listed in the top left corner of each plot. The red line indicates the 1:1 line

Species richness varied substantially among methods in the 2015 L3-WMP (Fig. 4). Species richness estimates were most similar between standard and laser quadrats (Fig. 4A), although the standard quadrat consistently captured more species than the laser quadrat. Species richness estimates were higher for point contact (Fig. 4B) and line intercept (Fig. 4C) methods relative to the standard quadrat. Richness estimates were relatively comparable between point contact and line intercept methods, with line intercept methods capturing consistently more species than point contact, irrespective of the species pool (Fig. 4D). Compared to laser quadrat, more species (2–3 times as many) were captured by both point contact (Fig. 4E) and line intercept (Fig. 4F) methods.

**Fig. 5:**
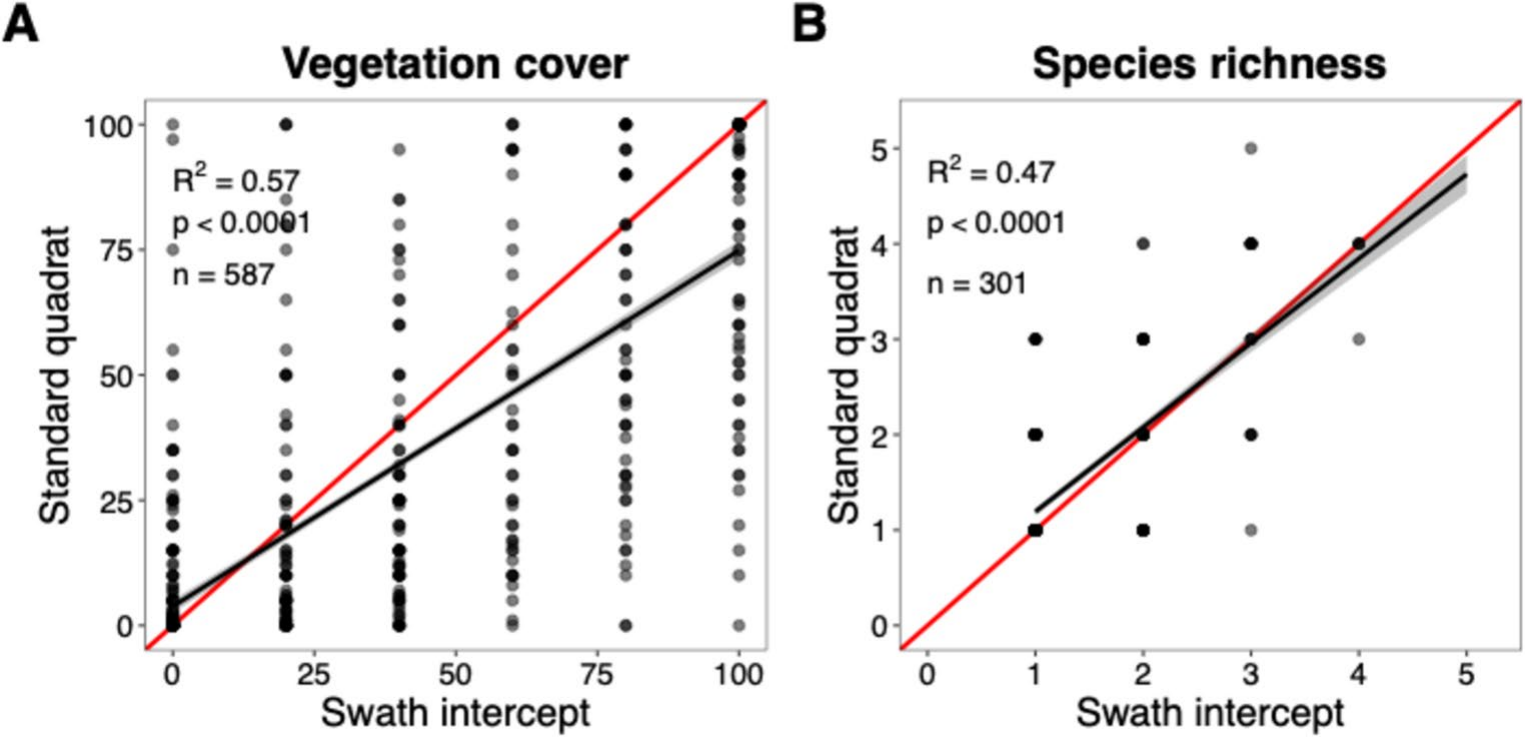
Methods comparison of vegetation cover and species richness for SONGS MMP program. Vegetation cover (**A**) and species richness (**B**) estimates comparing standard quadrat to swath intercept. Points represent vegetation cover estimates from the same survey area using each method, the fitted regression line includes the 95th % confidence interval, and the fit of the line (R^2^), associated p-value, and the sample size (n) are listed in the top left corner of each plot. The red line indicates the 1:1 line. In panel B, multiple points are overlapping. Darker shaded points on each panel represent higher density occurrences

**Fig. 6:**
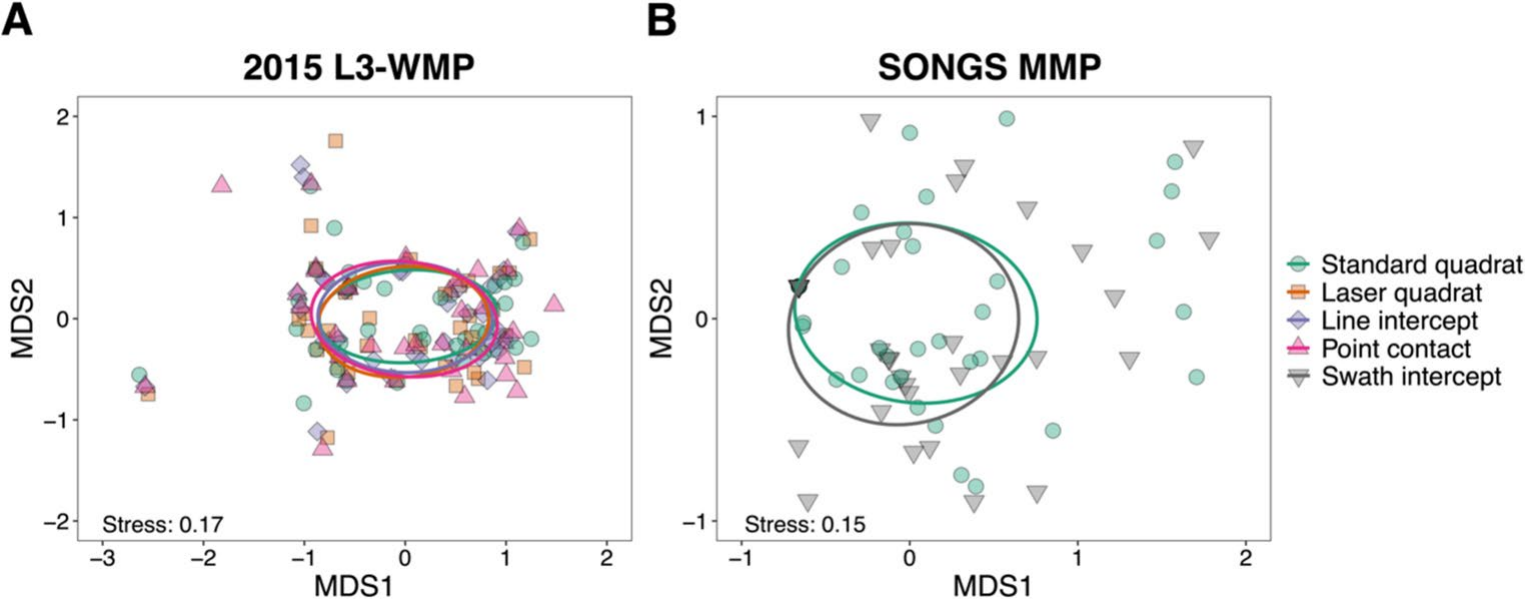
Methods comparison of community composition. Vegetation community composition comparisons across methods for the (**A**) 2015 L3-WMP (standard quadrat, laser quadrat, point contact, line intercept) and (**B**) SONGS MMP programs (standard quadrat, swath intercept). Points represent the community composition from the same survey area and method. Colors and shapes denote the method. Standard error ellipses represent 95% confidence intervals relative to the mean centroid for each method

For the SONGS MMP data, variation in vegetation cover and species richness estimates may be due to the fundamentally different survey protocols and area evaluated by the two methods (standard quadrat and swath intercept; Fig. 5A, B). For vegetation cover, agreement between the two methods decreased with increasing cover, the opposite pattern from what was observed by the 2015 L3-WMP program. However, there was strong agreement in estimates of species richness between the two methods, where these methods produce a linear relationship very close to 1:1 with a R^2^ of 0.47 (Fig. 5B). The inflection point for the SONGS MMP methods compared was at 20%, whereas most of the 2015 L3-WMP methods compared had inflection points where total vegetation cover was at or above 50% (Table 2). Community composition did not vary by method for data collected from either the 2015 L3-WMP (Fig. 6 A; F_3,144_ = 0.303; *p* = 1.0) or SONGS (Fig. 6B; F_1,88_ = 2.03; *p* = 0.078) programs.

### Survey Decisions

Adjusting survey decisions post-hoc within programs demonstrated how important the standardization of survey decisions is for comparisons across methods.

#### Bounding

As expected, unbounded vegetation cover estimates were greater than adjusted vegetation cover estimates, especially as cover increased (Fig. S1). For the 2015 L3-WMP data, there were instances where the unbounded vegetation cover estimates were lower than the adjusted unbounded estimates. This pattern is most likely due to the use of binned data, where the mid-point of the bin was used to plot cover.

#### Nomenclature

**Fig. 7:**
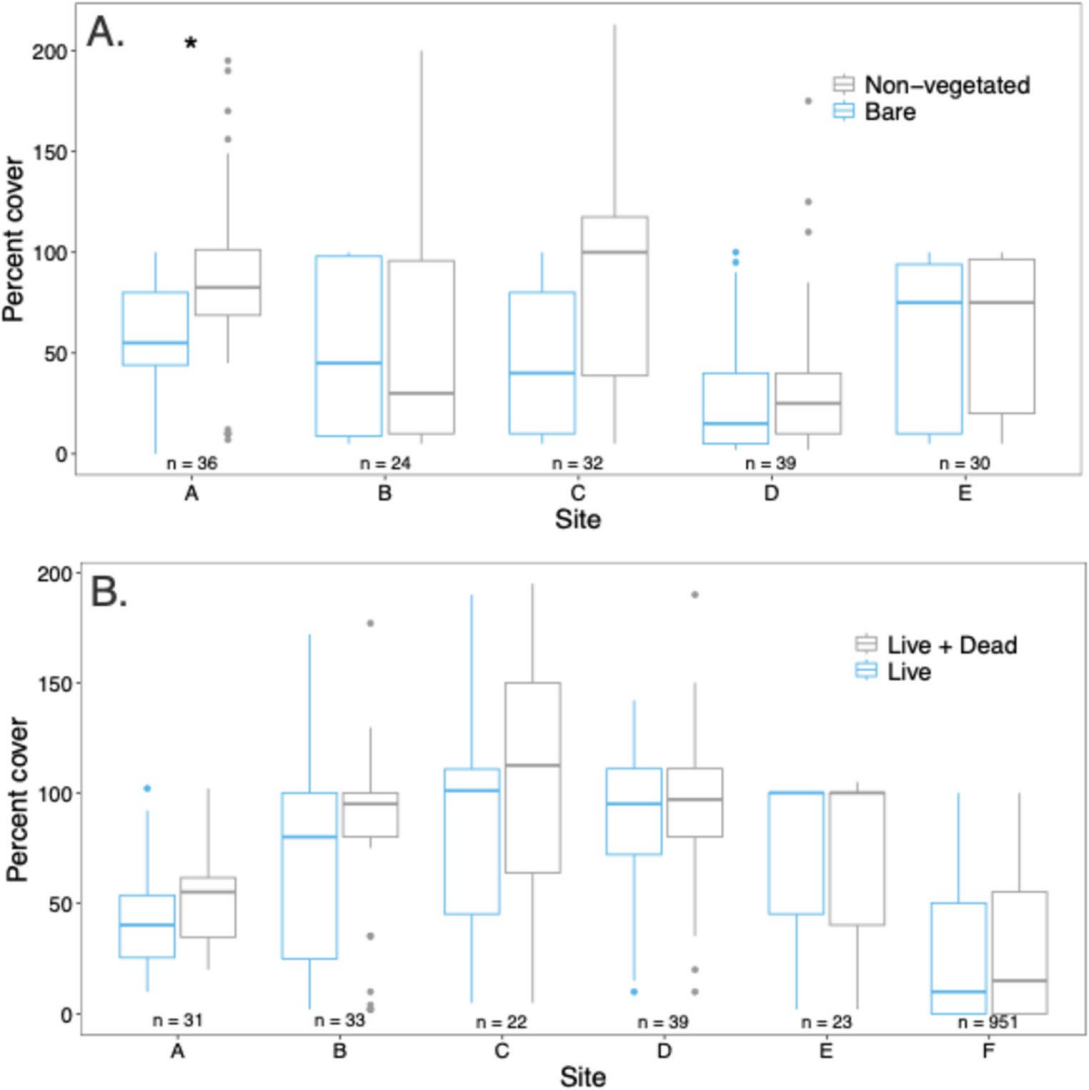
Comparison of unbounded non-vegetated cover and live vs. dead survey decisions within standard quadrats. (**A**) Estimates of percent cover for the bare subcategory and overall non-vegetated category. (**B**) Estimates of percent cover for live species states plus dead states and only live state estimates across six sites. Boxplots show median (bold lines) and interquartile range (boxes), with outliers greater than 1.5 × IQR (whiskers). Asterisks indicate significant differences among between groups within a site (α = 0.05). Sites A-E are from the EMPA program, whereas site F is from the SONGS MMP program. Sample sizes indicate the number of replicates per site. All measurements are from a standard quadrat

When non-vegetated categories were allowed to exceed 100%, we found that comparing the bare category to the bare + non-vegetated category (bare + wrack + algae + etc.) was comparable at four of the five sites (Fig. 7A). Percent cover was significantly higher for site A (F_1, 69_ = 9.94, *p* = 0.002) when bare and non-vegetated cover classes were grouped.

#### Plant state

Grouping live and dead plant states versus only using live vegetation did not affect estimates of vegetation cover (Fig. 7B).

#### Binning

**Fig. 8:**
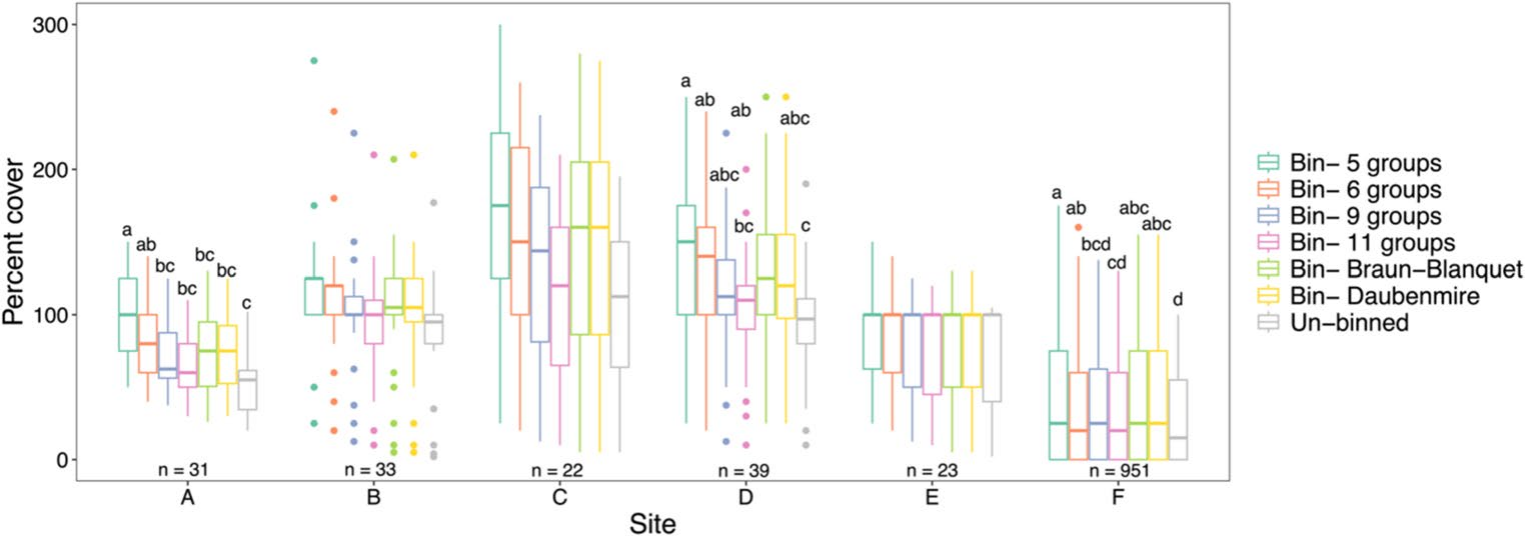
Comparison of the binned vegetation cover survey decision from the standard quadrats. Estimates of percent cover binned into eight categories – 5 groups, 6 groups, 9 groups, 11 groups, Braun-Blanquet, Daubenmire, and un-binned. Sites A-E are from the EMPA program whereas site F is from the SONGS MMP program. Lower-case letters indicate significant differences among bins within a site (α = 0.05). Boxplots show median (bold lines) and interquartile range (boxes), with outliers greater than 1.5 × IQR (whiskers). Sample sizes indicate the number of replicates per site. All measurements are from a standard quadrat

Although results varied by site, increasing the number of evenly-spaced bins more closely approximated the un-binned, vegetation cover measurements, and the 11-bin approach best approximated the unbinned data. (Fig. 8). Uneven binning methods (Daubenmire, Braun-Blanquet methods) were equivalent to the evenly binned approach with the same number of bins (6 groups; Fig. 8).

## Discussion

**Fig. 9:**
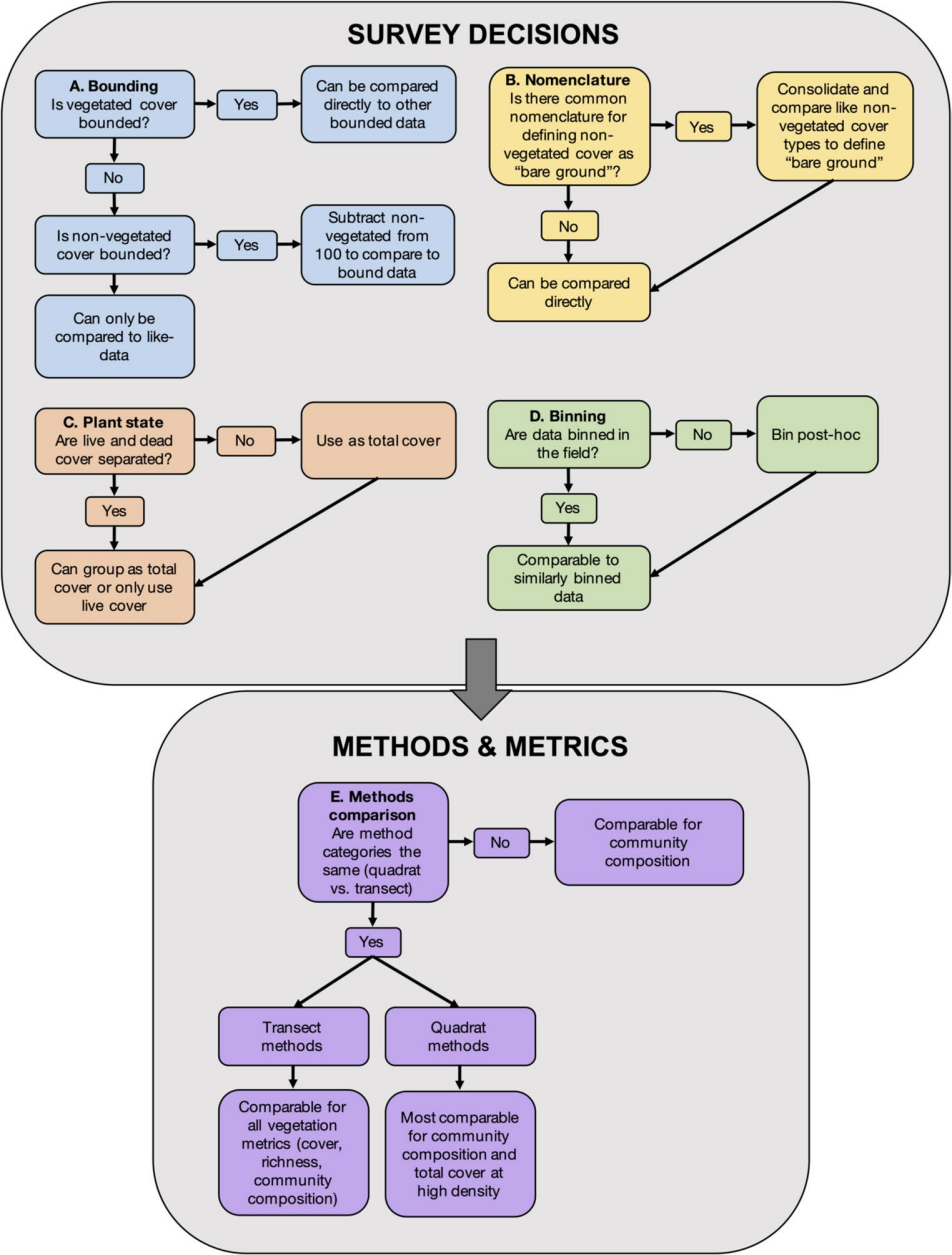
Decision tree for determining whether data collected with different underlying survey decisions, methods, and metrics are comparable. Decision tree for determining whether data collected with different survey decisions, including those related to (**A**) bounding, (**B**) nomenclature, (**C**) plant state, and (**D**) binning, and (**E**) methods and metrics are comparable

Although many nationwide (e.g., NARS, NERRS) and statewide (e.g., California, Louisiana) programs have initiated substantive efforts to develop standardized site-intensive (i.e., Level 3) assessment methods and a framework for their application in coastal wetlands (California Wetlands Monitoring Workgroup, 2010; Johnston et al., 2021), evaluation of monitoring methods is necessary to continue progressing towards a comprehensive and cohesive nationwide and statewide monitoring strategy. This project compared vegetation monitoring methods and survey decisions within programs with distinct goals to support future synthesis among disparate monitoring programs. Similar to other regions and studies (Damgaard, 2014; Godínez-Alvarez et al., 2009; Raposa et al., 2020), comparisons among methods, with specific caveats discussed below, were possible for a variety of vegetation metrics with consistent over- and under-estimation for certain methods. We found that vegetation data collected within a type of method (e.g., all transect or all quadrat) was generally more comparable than data collected across method types (e.g., transect and quadrat). Whether the methods were comparable depended on three factors: (1) method category (transect vs. quadrat), (2) the total cover (high vs. low), and (3) the type of vegetation metrics (percent cover, species richness, or community composition). Surprisingly, the standardization across three of the four survey decisions within a program was not key to the ability to compare vegetation metrics. However, if survey decisions are more similar among programs, the data may require less manipulation and therefore have higher quality assurance. Therefore, we would only recommend consolidating data types across programs if similar method types and survey decisions were used. We synthesize the method comparisons and survey decisions to draw six overarching conclusions (in **bold**) that could be applied to future comparative syntheses that, unlike this study, pool data across programs. We also provide a decision tree to aid in determining when data collected with different underlying survey decisions, methods, and metrics are comparable, which leads to our recommendation of standardized, site-intensive vegetation monitoring moving forward (Fig. 9).

1. **Transect-based methods are more comparable to one another than comparisons between transect- and quadrat-based methods.** Transect-based protocols specify precisely where to survey as opposed to subjective visual cover estimates used by haphazardly placed, quadrat-based methods (Fig. 9E; Dethier et al., 1993; Godínez-Alvarez et al., 2009; Raposa et al., 2020). Laser quadrat methods are similarly objective because they are prescriptive in the precise location of the visual estimates. Other quadrat methods are not prescriptive but rather estimate a larger area, which contributes to their consistent over- and under-estimation of percent cover relative to transect-based methods. This bias was predictable and therefore could be used in the future to compare disparate methods across programs. Further analysis should be conducted at the individual species level to see if trends are similar.
2. **The density of cover influences the comparability of methods.** The standard quadrat method tended to overestimate cover at the lower cover end relative to other methods (i.e., point contact, laser quadrat, line intercept), with the exception of swath intercept. This overestimation of cover occurred when vegetation was sparse, as small patches of plants are often visually overrepresented. This pattern has been observed in other studies across a range of ecosystems (e.g., river plain, Floyd & Anderson, 1987; boreal forest, Milberg et al., 2008). Therefore, when vegetation was dense (high cover), results were more similar among methods (Fig. 9E). The exact mechanism of this overestimation cannot be determined but could be observers’ abilities to estimate sparse vegetation cover or could be an artifact of how standard quadrat methodoloy records unvegetated cover (i.e., treats unvegetated cover and vegetated cover equally). This finding is especially important when considering monitoring newly restored or degraded sites, or areas with naturally low cover. Due to potential overestimation, methods are likely less comparable in these low cover systems and comparisons across methods and programs should be done with caution.
3. **Method comparability depends on the vegetation metric (e.g.**,** total percent cover**,** species richness**,** community composition).** Of the three targeted vegetation metrics, community composition was similar across all methods (Fig. 9E), despite the nuances between transect and quadrat methods described above. In contrast, patterns in relative over- and under-estimation for species richness and total vegetation cover depended on method. Species richness varied by survey method, with standard quadrats detecting higher richness relative to laser quadrats, and detecting lower richness relative to point contact, line intercept, and swath intercept methods in high cover plots (Figs. 4 and 5). In general, quadrat-based methods generally found fewer species relative to transect-based methods, suggesting that rarer species were less likely to be captured with quadrat-based methods (Fig. 9E). Species richness classically scales with survey area and effort (Godínez-Alvarez et al., 2009; Gotelli & Colwell, 2001), so differences in area sampled among methods can affect estimates of species richness. In our study, within a site, transect-based methods captured a larger area than quadrat-based methods, which may explain the greater species richness estimates produced by transect-based methods. Interestingly, these results contrast with terrestrial systems (e.g., rangelands, prairies, desert shrublands), where transect-based methods generally underestimate species richness due to smaller total area sampled and spatial autocorrelation (Godínez-Alvarez et al., 2009; Goslee, 2006; Stohlgren et al., 1998).
  Results may also differ from terrestrial systems due to the lower overall species pool in coastal wetland ecosystems. Species richness estimates may be more consistent among methods in low-richness systems. Indeed, in our study, we found that methods were most aligned for estimating species richness for the SONGS MMP, which measured a newly restored wetland with a smaller species pool. The visibility of plant species in a newly restored marsh with sparse canopy cover is higher than one that has a fully developed marsh canopy filled with different species and higher complexity in layers of vegetation, which may explain the agreement among results for the sites with lower overall canopy cover. Accounting for differences in area, sampling effort and background species pool with species rarefaction or accumulation curves could support standardization across methods and sites(Gotelli & Colwell, 2001) by assessing species richness estimates to the lowest sample size or area. In addition, there are other vegetation metrics that were not considered as part of this project, such as individual species cover, species diversity, and the ratio of native to non-native plant species (Noss, 1990; Stern et al., 2021). These types of metrics could also influence the comparability of methods, and therefore further studies that test these other metrics are necessary to more fully understand how comparability varies across methods and metrics. To validate the relevance of this study’s findings on systems outside our region or climate regime, similar investigations should be conducted with differing physical and climatic forcing.

(4) **Method comparability depends on the decision to bound vegetated and non-vegetated cover.** Whether or not a program bounds vegetated and non-vegetated cover influences data comparability (Fig. 9A). While our study focused on comparing total vegetated cover estimates across methods within programs, this is a critical metric to eventually compare across programs as well One strategy to adjust unbounded vegetated estimates for comparison to bounded estimates is to subtract the amount of estimated non-vegetated cover from 100% (total adjusted vegetation = 100 - non-vegetated cover). However, this adjustment requires a measurement of non-vegetated cover that does not exceed 100% (Fig. 9A). In the future, we recommend all programs first collect an estimate of total vegetated cover and non-vegetated cover out of 100% before measuring unbounded estimates. This approach supports comparison among programs and methods, while also enabling accurate assessment of species-specific and non-vegetated contributions to percent cover. Additionally, capturing plot photos can be valuable to help interpret findings, troubleshoot discrepancies, and provide a reference through time (NERRS, 2025).
(5) **Surveyor decisions**,** such as prescriptive non-vegetated nomenclature**,** categorizing live and dead vegetation**,** and binning data**,** do not greatly influence estimates of cover**,** but rather increase the amount of data analysis decisions.** We compared surveyor decisions from two programs and six sites, and at the majority of sites, surveyor decisions did not influence overall estimates of vegetation cover. One survey decision, whether or not the data was binned, did influence how comparable cover estimates were between methods depending on the final selection of bins. At three of the six sites, cover estimates varied based on the number of bins, where at all three sites, the unbinned category estimated cover lower than the smallest bin category (5 bins). However, by increasing the number of evenly-spaced bins (e.g. 5%), surveyors can more closely approximate the un-binned category. This lack of standardization prior to monitoring can compromise analysis and synthesis of estuary condition data, especially on a larger spatial scale spanning multiple monitoring programs.
  Resolving differing survey decisions increased post-processing time needed to make the data comparable. For example, when trying to post-calculate a total vegetated cover for the EMPA program, the subtraction correction not only depended on whether the non-vegetated category was unbounded, but also on consistent nomenclature for vegetation categories and definition of non-vegetated cover. The terminology associated with “bare ground” is highly variable between programs, with some incorporating all non-vegetated cover, and others separating out metrics such as terrestrial debris, wrack, or trash (Helm & Mead, 2004; Raposa et al., 2020). Consistency in nomenclature is important, and evaluations within and across programs with differing terminology can be challenging and may require consolidation of like non-vegetated cover types to support comparison (Fig. 9B). Furthermore, clear attribution of whether cover should be assigned to total vegetated or total non-vegetated cover is important for potential comparison across studies.
  Overall, we found many trade-offs to these decisions, and when decisions were consistent among datasets, the comparability of the data increased. In most cases, the more the data was parsed during collection, the easier it was to lump together later. For example, although the results suggested similar findings between incorporation of both live and dead vegetation (versus just live vegetation), site-specific monitoring needs suggest that separately monitoring live and dead vegetation states may provide additional insight into marsh function and processes (e.g., primary production, decomposition, nutrient cycling), as well as the ability to track succession or restoration success (McCall & Pennings, 2012; Stagg & Mendelssohn, 2010). Therefore, if live and dead estimates were collected in the field, then these estimates could be combined as “total vegetated cover” later during analysis (Fig. 9C). Additionally, if estimates of cover are measured as exact cover, then cover estimates can be binned during analysis (Fig. 9D). Arguments against this method of estimating exact cover suggest that when visual estimates of cover are based on cover classes, precision is reduced but accuracy improves (Bonham et al., 2004; Helm & Mead, 2004). When there are broadly defined cover classes, there is less chance for consistent human error in assigning the cover class (Bonham et al., 2004; Daubenmire, 1959). Therefore, it is critical in the program metadata or in the establishment of a monitoring program to accurately choose terminology and comprehensive Standard Operating Procedures (SOPs) that outline these decisions.

(6) **Data consolidation of disparate methods should be done with caution for historical data**,** and programs should prioritize standardization moving forward.** We examined how methods could vary within programs and then assessed whether those patterns were similar across programs. In general, we found that vegetation collected within a type of method (e.g., transect or quadrat) was generally more comparable than that collected across method types (e.g., transect vs. quadrat; Fig. 9E); therefore, we would recommend only consolidating data types across programs in which similar method types were used. Although there were consistent method biases when estimating cover, we did not directly test whether results could be converted to overcome biases and have more comparable data. Further studies that directly test data consolidation across programs are necessary. Overall, when attempting to combine datasets, tradeoffs need to be weighed between the amount of uncertainty in method comparability and the benefits of data comparison. If comparisons across historical datasets are needed, we encourage programs to consider focusing on community composition using multivariate analyses, as this metric was comparable across all methods in this study (Fig. 9E). Ordination plots (e.g., nMDS) are useful to evaluate and visualize the relationships between variables or sites and can be particularly helpful for comparing different methods or treatments (Kenkel & Orlóci, 1986; Lepš & Hadincová, 1992; Ruokolaninen & Salo, 2006). That said, it is important to consider and potentially analyze within other site-specific factors beyond method (e.g., elevation, freshwater input, etc.), that could be driving community differences. Moving forward, we recommend standardizing both survey methods and decisions to collect more robust, comparable datasets.

### Other Considerations

When standardizing methods across programs, there are other decisions to consider. First, there will always be an observer bias, where individual surveyors can bias specific plants and/or values (Milberg et al., 2008). For example, when estimating cover, surveyors tend to estimate cover to the nearest 5%, where an observer is more likely to estimate cover at 75% rather than 74% or 76%. These types of biases could impact the data if binning occurs during analysis. For example, if the bins are set at 50–74% and 75–99%, there may be a consistent bias towards the second bin due to observers more likely selecting 75% over 74%. Acknowledging the observer bias in the dataset is critical during analysis. Second, replication and scale could influence both species richness and species composition (Gibbons & Freudenberger, 2006). As scale and replication increase, surveyors are more likely to find rarer species. Therefore, both scale and replication should be considered in the analysis. Depending on the monitoring question, replication should be standardized to make accurate assessments across sites. Third, the amount of time, effort, and cost of each method should be considered when selecting monitoring approaches. In selecting an approach, the amount of personnel and budget could impact method selection, and resources, such as Johnston et al. (2021), could be used to inform decisions. Lastly, integrated metrics can help overcome standardization issues. For example, a recently developed metric called the unvegetated - vegetated marsh ratio (UVVR; Ganju et al., 2022) uses a combination of remote sensing and field-based methods to assess overall marsh cover. Utilizing remote sensing tools allows researchers to track multiple sites and regions with a single data set. This increases efficiencies and standardizes the processes across agencies and partners.

### Monitoring Recommendations

Overall, our results agree with earlier studies both in marshes and other ecosystems (Damgaard, 2014; Floyd & Anderson, 1987; Godínez-Alvarez et al., 2009; Raposa et al., 2020) that show data from these types of methods are highly repeatable and comparable. However, to make robust comparisons within and across regions and over the long-term, a single method should be selected. When deciding between the best monitoring approach, the choice of method depends on the specific monitoring questions and/or goals of the program. To be successful in comparing monitoring methods and the associated data, programs need to have clear and robust programmatic guidance and metadata. Therefore, we recommend that monitoring programs include extensive programmatic guidance that defines terminology, depicts the sampling design, details the Standard Operating Protocols (SOPs), and provides extensive metadata associated with the datasets. This extensive guidance will help future practitioners compare across programs when necessary.

Depending on site conditions and program goals, standard quadrat and line intercept methods both offer relatively strong comparability for total cover and richness metrics (Fig. 9E). Although there was some agreement among all methods for total vegetation cover, especially for the higher cover estimates, and community composition, the laser quadrat and point-contact methods consistently underestimated species richness. However, if the standard quadrat method is used, additional site surveys for non-dominant or rare plant species may also be warranted. For all methods, developing a species list through site searches adjacent to the transects would supplement species richness for low-cover species by habitat or site.

We recommend standardizing survey decisions to collect the most complete and consistent data. First, we recommend beginning the quadrat-based survey with an initial estimate of bounded vegetated cover and non-vegetated cover, with clear definitions of these categories. Here, vegetated and non-vegetated cover should equal 100%. By including these metrics first, this approach negates the difference between unbounded and bounded cover estimates and allows for cross-program comparisons of total vegetated cover. We define vegetated cover as the area of the plot with upright, rooted, live or dead plants. Non-vegetated cover is the amount of the plot not covered with vegetated material and can include other cover types, such as bare ground, algae, detritus, or debris. The common cover type that varied the most among survey methods was non-vegetated cover. The amount of non-vegetated area can be a useful indicator of stress to a marsh, particularly from pollution, drought, sea-level rise, and excessive inundation (Ganju et al., 2017; Raposa et al., 2016). Additionally, monitoring changes in non-vegetated areas over time can provide insights into the recovery processes and successional stages of vegetation in restored or disturbed marshes (Crain et al., 2008; Hughes et al., 2009). Non-vegetated cover should be a core metric of monitoring programs concerned with these issues (Neckles et al., 2015; Raposa et al., 2020).

We also recommend measuring unbounded estimates of species cover once the bounded cover has been assessed. Unbounded total cover captures the multi-layered structure of vegetation and supports more accurate assessment of species-specific contributions to percent cover. This approach is also important in habitats with complex vertical stratification (Keer & Zedler, 2002) or for research questions where function is related to complexity (e.g., habitat provision for birds; Zedler, 1993). In addition, unbounded cover provides relative magnitude of species contribution to the overall vegetation cover, which can be useful for understanding species dominance and community composition. However, because cover percentages can exceed 100% with unbounded data, this method may overestimate the actual ground cover, which can complicate comparisons between plots and can be more complex to analyze and interpret, especially when comparing sites or tracking changes over time. To compensate for this issue, we recommend first estimating bounded cover estimates for vegetated and non-vegetated cover. The use of electronic data entry in the field using programs such as ESRI’s Survey123 can help ensure that estimates of bounded and unbounded cover are consistent (that individual species cover adds up at least to the bounded estimate of vegetation cover). This method allows field crews to quickly detect inconsistencies while still in the field.

Finally, we encourage all monitoring programs to host field training and calibration exercises among monitoring teams and sites to minimize the potentially large differences in specific method application, observer bias, and data collection strategies. Data collection techniques associated with the SOPs should also be standardized across the monitoring program and data collection efforts. Within a program, protocols should also be standardized over time to support collection of consistent time series.

This project represents an effort to improve consistency in site-intensive vegetation monitoring approaches. The goal of standardization is to have methods remain project-specific, yet also be diagnostic of broader restoration performance, regulatory compliance, and the health and condition of systems in the region.

## Supplementary Information

Below is the link to the electronic supplementary material.

Supplementary Material 1 (DOCX 195 KB)

## Data Availability

The datasets from the 2015 L3-WMP and SONGS Programs are available from the corresponding author on request. The EMPA program datasets are available online via the EMPA data portal - https://empa.sccwrp.org.

## References

[CR1] Alam, S. M. R., & Hossain, M. S. (2021). A rule-based classification method for mapping saltmarsh land-cover in South-Eastern Bangladesh from Landsat-8 OLI. *Canadian Journal of Remote Sensing,**47*(3), 356–380. 10.1080/07038992.2020.1789852

[CR2] Ambrose, R. F., & Diaz, N. (2008). Pre-spill Assessments of Coastal Habitat Resources. 1: Development of Protocols. 2: Quick Response Protocols. *Report to the California Department of Fish and Game Office of Spill Prevention and Response* https://nrm.dfg.ca.gov/FileHandler.ashx?DocumentID=19923&inline

[CR3] U.S. Army Corps of Engineers (2010). Chapter 2: *Hydrophytic Vegetation. Powerpoint by Steve Eggers. Retrieved December 12, 2025 from* https://www.mvp.usace.army.mil/portals/57/docs/regulatory/regulatorydocs/hydrophyticvegetationapril2010.pdf

[CR4] Barbier, E. B., Hacker, S. D., Kennedy, C., Koch, E. W., Stier, A. C., & Silliman, B. R. (2011). The value of estuarine and coastal ecosystem services. *Ecological Monographs,**81*(2), 169–193. 10.1890/10-1510.1

[CR5] Bonham, C. D., Mergen, D. E., & Montoya, S. (2004). Plant Cover Estimation: A Contiguous Daubenmire Frame. *Rangelands*, *26*(1), 17–22. 10.2111/1551-501X(2004)26%255B17:PCEACD%255D2.0.CO;2.

[CR6] Braun-Blanquet, J. (1925). J. Zur Wertung der Gesellschaftstreue in der Pflanzensoziologie. *Vierteljahrsschrift Der Naturforschenden Gesellschaft in Zürich*, *70*, 122–149.

[CR7] Cahoon, D. R., Lynch, J. C., Roman, C. T., Schmit, J. P., & Skidds, D. E. (2019). Evaluating the relationship among wetland vertical development, elevation capital, sea-level rise, and tidal marsh sustainability. *Estuaries and Coasts,**42*(1), 1–15. 10.1007/s12237-018-0448-x

[CR8] California Wetlands Monitoring Workgroup (2010). *Tenets of a State Wetland and Riparian Monitoring Program (WRAMP). Pp. 75.* 75 pp. https://www.mywaterquality.ca.gov/monitoring_council/wetland_workgroup/wramp/

[CR9] Coastal Protection and Restoration Authority (CPRA) of Louisana (2022). *Coastwide Reference Monitoring System-Wetlands Monitoring Data. Retrieved from Coastal Information Management System (CIMS) database. Retrieved August 12, 2025, from* https://cims.coastal.la.gov

[CR10] R Core Team (2024). *R: A language and environment for statistical computing. R Foundation for Statistical Computing* [Computer software]. https://www.R-project.org/

[CR11] Crain, C. M., Albertson, L. K., & Bertness, M. D. (2008). Secondary succession dynamics in estuarine marshes across landscape-scale salinity gradients. *Ecology,**89*(10), 2889–2899. 18959326 10.1890/07-1527.1

[CR12] Dahlgren, R. A., Singer, M. J., & Huang, X. (1997). Oak tree and grazing impacts on soil properties and nutrients in a California oak woodland. *Biogeochemistry*, *39*(1), 45–64. 10.1023/A:1005812621312

[CR13] Dale, J., Burnside, N. G., Hill-Butler, C., Berg, M. J., Strong, C. J., & Burgess, H. M. (2020). The use of unmanned aerial vehicles to determine differences in vegetation cover: A tool for monitoring coastal wetland restoration schemes. *Remote Sensing,**12*(24), Article 4022. 10.3390/rs12244022

[CR14] Damgaard, C. (2014). Estimating mean plant cover from different types of cover data: A coherent statistical framework. *Ecosphere*, *5*(2), 1–7. 10.1890/ES13-00300.1

[CR15] Daubenmire, R. F. (1959). Canopy coverage method of vegetation analysis. *Northwest Science*, *33*, 43–64.

[CR16] Department of Agriculture, U. S., & U.S. Department of the Interior. (1996). &. *Sampling Vegetation Attributes. Interagency Technical Reference. Cooperative Extension Service. BLM/RS/ST*-96/002 + 1730. *Retrieved on December 12, 2025 from https://www.nrcs.usda.gov/sites/default/files/2022-09/stelprdb1044175.pdf*

[CR17] Dethier, M., Graham, E., Cohen, S., & Tear, L. (1993). Visual versus random-point percent cover estimations: Objective is not always better. *Marine Ecology Progress Series*, *96*, 93–100. 10.3354/meps096093

[CR18] EMPA (2024). *California Estuary Marine Protected Area Monitoring Program. Standard Operating Procedure #11: Vegetation Surveys.* https://ftp.sccwrp.org/pub/download/PROJECTS/EMPA/sopPDFs/SOPs/update0824/SOP11_veg_Aug2024.pdf

[CR19] U.S. Environmental Protection Agency (2024). *National Aquatic Resource Surveys. National Wetland Condition Assessment 2021. Retrieved August 12, 2025, from* https://www.epa.gov/national-aquatic-resource-surveys/data-national-aquatic-resource-surveys

[CR20] Floyd, D. A., & Anderson, J. E. (1987). A Comparison of Three Methods for Estimating Plant Cover. *The Journal of Ecology*, *75*(1), 221. 10.2307/2260547

[CR21] Ganju, N. K., Defne, Z., Kirwan, M. L., Fagherazzi, S., D’Alpaos, A., & Carniello, L. (2017). Spatially integrative metrics reveal hidden vulnerability of microtidal salt marshes. *Nature Communications*. 10.1038/ncomms14156 10.1038/ncomms14156 PMC5264011 28112167

[CR22] Ganju, N. K., Couvillion, B. R., Defne, Z., & Ackerman, K. V. (2022). Development and Application of Landsat-Based Wetland Vegetation Cover and UnVegetated-Vegetated Marsh Ratio (UVVR) for the Conterminous United States. *Estuaries and Coasts*, *45*(7), 1861–1878. 10.1007/s12237-022-01081-x

[CR23] Gibbons, P., & Freudenberger, D. (2006). An overview of methods used to assess vegetation condition at the scale of the site. *Ecological Management & Restoration*. 10.1111/j.1442-8903.2006.00286.x

[CR24] Godínez-Alvarez, H., Herrick, J. E., Mattocks, M., Toledo, D., & Van Zee, J. (2009). Comparison of three vegetation monitoring methods: Their relative utility for ecological assessment and monitoring. *Ecological Indicators,**9*(5), 1001–1008. 10.1016/j.ecolind.2008.11.011

[CR25] Goslee, S. C. (2006). Behavior of Vegetation Sampling Methods in the Presence of Spatial Autocorrelation. *Plant Ecology*, *187*(2), 203–212. 10.1007/s11258-005-3495-x

[CR26] Gotelli, N. J., & Colwell, R. K. (2001). Quantifying biodiversity: Procedures and pitfalls in the measurement and comparison of species richness. *Ecology Letters,**4*(4), 379–391. 10.1046/j.1461-0248.2001.00230.x

[CR27] Hartig, F. (2002). *Residual Diagnostics for Hierarchical (Multi-Level / Mixed) Regression Models. R package. Version 0.4.6.* Https://CRAN.R-project.org/package=DHARMa (Version 0.4.6) [R package]. https://CRAN.R-project.org/package=DHARMa

[CR28] Helm, D. J., & Mead, B. R. (2004). Reproducibility of vegetation cover estimates in south-central Alaska forests. *Journal of Vegetation Science,**15*(1), 33–40. 10.1111/j.1654-1103.2004.tb02234.x

[CR29] Hughes, R., Fletcher, P., & Hardy, M. (2009). Successional development of saltmarsh in two managed realignment areas in SE England, and prospects for saltmarsh restoration. *Marine Ecology Progress Series*, *384*, 13–22. 10.3354/meps08027

[CR30] Johnston, K., Medel, I., Anderson, S., Abbott, R., & Stein, E. (2015). Regional Monitoring Report for Southern California Coastal Wetlands: Application of the USEPA Three-Tiered Monitoring Strategy. *Prepared by The Bay Foundation for the United States Environmental Protection Agency*, 100 pp.

[CR31] Johnston, K., Grubbs, M., Whitcraft, C., Crooks, J., Uyeda, K., & Enyart, C. (2020). Evaluation and Regional Comparison of USEPA Intensive, Level-3 Monitoring: Consolidating Coastal Wetland Datasets and Programs. *Prepared by The Bay Foundation and Partners for the United States Environmental Protection Agency*, 34 pp.

[CR32] Johnston, K., Medel, I., Whitcraft, C., Crooks, J., Grubbs, M., Anderson, S., Uyeda, K., Stein, E., & Enyart, C. (2021). California Estuarine Wetland Monitoring Manual (Level 3) V2. *Prepared by The Bay Foundation and Partners for the United States Environmental Protection Agency*, 385 pp.

[CR33] Keer, G. H., & Zedler, J. B. (2002). Salt marsh canopy architecture differs with the number and composition of species. *Ecological Applications*, *12*(2), 456–473. 10.1890/1051-0761(2002)012%255B0456:SMCADW%255D2.0.CO;2.

[CR34] Kenkel, N. C., & Orlóci, L. (1986). Applying metric and nonmetric multidimensional scaling to ecological studies. *Ecology*, *67*(4), 919–928.

[CR35] Kennedy, K. A., & Addison, P. A. (1987). Some considerations for the use of visual estimates of plant cover in biomonitoring. *The Journal of Ecology,**75*(1), 151. 10.2307/2260541

[CR36] Kent, M., & Coker, P. (1992). *Vegetation description and analysis: A practical approach*. CRC Press.

[CR37] Lavorel, S. (2013). Plant functional effects on ecosystem services. *Journal of Ecology*, *101*(1), 4–8. 10.1111/1365-2745.12031

[CR38] Lepš, J., & Hadincová, V. (1992). How reliable are our vegetation analyses? *Journal of Vegetation Science*, *3*(1), 119–124. 10.2307/3236006

[CR39] Lopes, C. L., Mendes, R., Caçador, I., & Dias, J. M. (2020). Assessing salt marsh extent and condition changes with 35 years of Landsat imagery: Tagus Estuary case study. *Remote Sensing of Environment,**247*, Article 111939. 10.1016/j.rse.2020.111939

[CR40] McCall, B. D., & Pennings, S. C. (2012). Geographic variation in salt marsh structure and function. *Oecologia,**170*(3), 777–787. 10.1007/s00442-012-2352-6 22614261 10.1007/s00442-012-2352-6

[CR41] Milberg, P., Bergstedt, J., Fridman, J., Odell, G., & Westerberg, L. (2008). Observer bias and random variation in vegetation monitoring data. *Journal of Vegetation Science*, *19*(5), 633–644. 10.3170/2008-8-18423

[CR42] Millennium Ecosystem Assessment. (2005). *Ecosystems and Human Well-being: Synthesis*. Island Press.

[CR43] Neckles, H. A., Lyons, J. E., Guntenspergen, G. R., Shriver, W. G., & Adamowicz, S. C. (2015). Use of structured decision making to identify monitoring variables and management priorities for salt marsh ecosystems. *Estuaries and Coasts,**38*(4), 1215–1232. 10.1007/s12237-014-9822-5

[CR44] NERRS (2025). *Estuarine Marsh Monitoring Standard Operating Procedure. National Estuarine Research Reserve System Technical Report.* https://cdmo.baruch.sc.edu/request-manuals/. *31pp.*

[CR45] Noss, R. F. (1990). Indicators for Monitoring Biodiversity: A Hierarchical Approach. *Conservation Biology*, *4*(4), 355–364. 10.1111/j.1523-1739.1990.tb00309.x

[CR46] Oksanen, J., Simpson, G., Blanchet, F., Kindt, R., Legendre, P., Minchin, P., O’Hara, R., Solymos, P., Stevens, M., Szoecs, E., Wagner, H., Barbour, M., Bedward, M., Bolker, B., Borcard, D., Carvalho, G., Chirico, M., De Caceres, M., Durand, S., & Weedon (2022). Vegan: Community Ecology. *R package*, *Version 2.6-4*. https://CRAN.R-project.org/package=vegan. https://CRAN.R-project.org/package=vegan (Version 2.6-4) [R package].

[CR47] Page, M., Schroeter, S., & Reed, D. (2022). Monitoring Plan for the SONGS’ Wetland Mitigation Project. *Prepared for the Staff of the California Coastal Commission*. https://marinemitigation.msi.ucsb.edu/sites/default/files/documents/wetland/wetland_mitigation_monitoring_plan_updated_2022-05-v02.pdf

[CR48] Page, H. M., Schroeter, S. C., Deza, A., Johnston, R., Smith, R. S., Beheshti, K. M., Reed, D. C., & Hoesterey, J. C. (2025). Lessons learned and value of early post-construction monitoring of a large tidal wetland restoration project. *Ecological Restoration,**43*(4), 261–276.

[CR49] Pendleton, L., Donato, D. C., Murray, B. C., Crooks, S., Jenkins, W. A., Sifleet, S., Craft, C., Fourqurean, J. W., Kauffman, J. B., Marbà, N., Megonigal, P., Pidgeon, E., Herr, D., Gordon, D., & Baldera, A. (2012). Estimating global “blue carbon” emissions from conversion and degradation of vegetated coastal ecosystems. *PLoS One,**7*(9), Article e43542. 10.1371/journal.pone.0043542 22962585 10.1371/journal.pone.0043542 PMC3433453

[CR50] Peter, C., Fischella, B., Raposa, K., Tyrrell, M., Allen, J., Mora, J., Goldstein, J., Feurt, C., Crane, L., & Burdick, D. (2020). A Guide to Integrate Plant Cover Data from Two Different Methods: Point Intercept and Ocular Cover. Report to the National Science Collaborative. Retrieved May 5, 2026 from https://nerrssciencecollaborative.org/sites/default/files/resources/Synthesizing%20Marsh%20Data_Plant%20Cover%20Integration%20Guide.pdf

[CR51] Raposa, K. B., Wasson, K., Smith, E., Crooks, J. A., Delgado, P., Fernald, S. H., Ferner, M. C., Helms, A., Hice, L. A., Mora, J. W., Puckett, B., Sanger, D., Shull, S., Spurrier, L., Stevens, R., & Lerberg, S. (2016). Assessing tidal marsh resilience to sea-level rise at broad geographic scales with multi-metric indices. *Biological Conservation,**204*, 263–275. 10.1016/j.biocon.2016.10.015

[CR52] Raposa, K. B., Kutcher, T. E., Ferguson, W., McKinney, R. A., Miller, K., & Wigand, C. (2020). Evaluation of Plot-Scale Methods for Assessing and Monitoring Salt Marsh Vegetation Composition and Cover. *Northeastern Naturalist*, *27*(1), 151. 10.1656/045.027.0113 10.1656/045.027.0113 PMC7863630 33551633

[CR53] Ruokolaninen, L., & Salo, K. (2006). Differences in performance of four ordination methods on a complex vegetation dataset. *Annales Botanici Fennici*, *43*(4), 269–275.

[CR54] SCWRP. (2018). *Wetlands on the Edge: The Future of Southern California’s Wetlands: Regional Strategy 2018*. California State Coastal Conservancy.

[CR55] Seabloom, E. W., & Valk, A. G. (2003). Plant diversity, composition, and invasion of restored and natural prairie pothole wetlands: Implications for restoration. *Wetlands*, *23*(1), 1–12. 10.1672/0277-5212(2003)023%255B0001:PDCAIO%255D2.0.CO;2.

[CR56] Sievert, C. (2020). *Interactive web-based data visualization with R, plotly, and shiny*. CRC Press, Taylor and Francis Group.

[CR57] Stagg, C. L., & Mendelssohn, I. A. (2010). Restoring Ecological Function to a Submerged Salt Marsh. *Restoration Ecology*, *18*(s1), 10–17. 10.1111/j.1526-100X.2010.00718.x

[CR58] Stern, J. L., Herman, B. D., & Matthews, J. W. (2021). Determining vegetation metric robustness to environmental and methodological variables. *Environmental Monitoring and Assessment,**193*(10), 647. 10.1007/s10661-021-09445-9 34519882 10.1007/s10661-021-09445-9

[CR59] Stohlgren, T. J., Bull, K. A., & Otsuki, Y. (1998). Comparison of rangeland vegetation sampling techniques in the Central Grasslands. *Journal of Range Management,**51*(2), 164. 10.2307/4003202

[CR60] Thomsen, A. S., Krause, J., Appiano, M., Tanner, K. E., Endris, C., Haskins, J., Watson, E., Woolfolk, A., Fountain, M. C., & Wasson, K. (2022). Monitoring vegetation dynamics at a tidal marsh restoration site: Integrating field methods, remote sensing and modeling. *Estuaries and Coasts,**45*(2), 523–538. 10.1007/s12237-021-00977-4

[CR61] Vittoz, P., & Guisan, A. (2007). How reliable is the monitoring of permanent vegetation plots? A test with multiple observers. *Journal of Vegetation Science*, *18*, 413–422.

[CR62] Watson, E. B., Wigand, C., Davey, E. W., Andrews, H. M., Bishop, J., & Raposa, K. B. (2017). Wetland loss patterns and inundation-productivity relationships prognosticate widespread salt marsh loss for Southern New England. *Estuaries and Coasts,**40*(3), 662–681. 10.1007/s12237-016-0069-1 30008627 10.1007/s12237-016-0069-1 PMC6040677

[CR63] Yeo, S., Lafon, V., Alard, D., Curti, C., Dehouck, A., & Benot, M.-L. (2020). Classification and mapping of saltmarsh vegetation combining multispectral images with field data. *Estuarine, Coastal and Shelf Science,**236*, Article 106643. 10.1016/j.ecss.2020.106643

[CR64] Zedler, J. B. (1993). Canopy Architecture of Natural and Planted Cordgrass Marshes: Selecting Habitat Evaluation Criteria. *Ecological Applications*, *3*(1), 123–138. 10.2307/1941796 27759219 10.2307/1941796

