# Supplementary material for: Comparison of Coastal Wetland Vegetation Assessment Methods in Southern California
Source: Estuaries Coast. 2026 Jul 20;49(5):162. doi: 10.1007/s12237-026-01779-2 (PMC13381379; doi:10.1007/s12237-026-01779-2)

# **Supplemental Information**

**Comparison of coastal wetland vegetation assessment methods in southern California**

**Authors:**

Janet B. Walker^1^, Rachel S. Smith^2^, Kathryn M. Beheshti^2^, Karina K. Johnston^2^, Christine R. Whitcraft^3^, Jeffrey A. Crooks^4^, Melodie Grubbs^5^, Henry M. Page^2^, Steve Schroeter^2^, Eric D. Stein^1^, and Kellie A. Uyeda^4, 6^

**Affiliations:**

^1^ Southern California Coastal Water Research Project, Costa Mesa, CA, USA

^2^ Marine Science Institute, University of California Santa Barbara, Santa Barbara, CA, USA

^3^ California State University, Long Beach, Long Beach, CA, USA

^4^ Tijuana River National Estuarine Research Reserve, Imperial Beach, CA, USA

^5^ Morro Bay National Estuary Program, Morro Bay, CA, USA

^6^ University of California San Diego, La Jolla, CA, USA

**Corresponding author:** Janet Walker, [janw@sccwrp.org](mailto:janw@sccwrp.org), ORCID: 0000-0001-7735-5879

June 30, 2026

# **Supplemental Fig. 1.** Comparison of unbounded and adjusted total vegetation cover for standard quadrats. Unbounded vegetation cover versus adjusted total vegetation cover using data from the (A) 2015 L3-WMP (100-non-vegetated) and (B) EMPA (100-bare) programs. Points represent vegetation cover estimates from the same plot using each method, the fitted regression line includes the 95th % confidence interval, the fit of the line (R2) is in the top left corner of each plot. The red line indicates the 1:1 line.
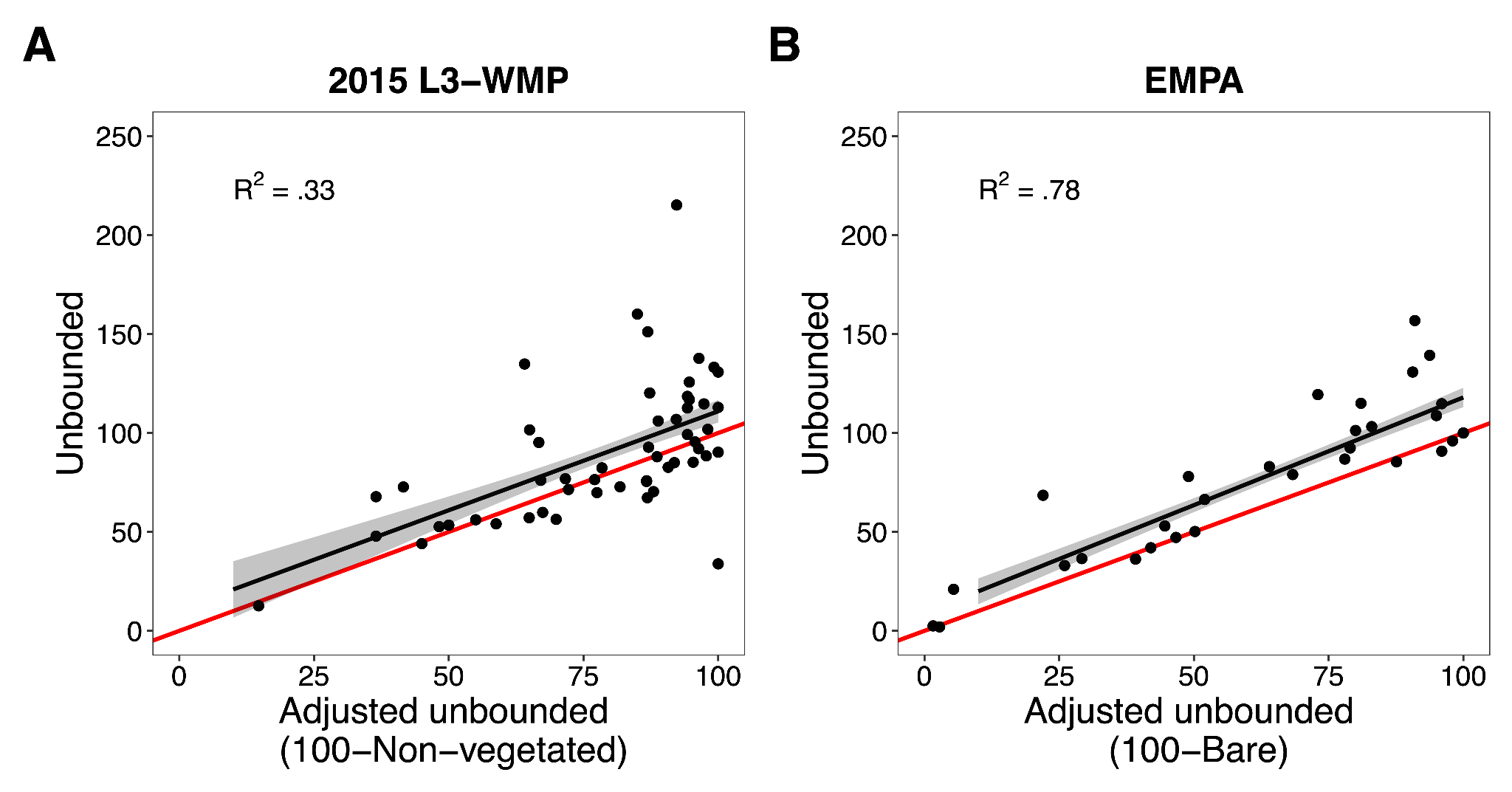

Supplement: Supplementary file 1 — Supplementary Material 1 (DOCX 195 KB) [file 12237_2026_1779_MOESM1_ESM.docx]
